# A positive feedback loop of CENPU/E2F6/E2F1 facilitates proliferation and metastasis via ubiquitination of E2F6 in hepatocellular carcinoma

**DOI:** 10.7150/ijbs.69495

**Published:** 2022-06-21

**Authors:** Yingyi Liu, Ye Yao, Bo Liao, Hao Zhang, Zhangshuo Yang, Peng Xia, Xiang Jiang, Weijie Ma, Xiaoling Wu, Chengjie Mei, Ganggang Wang, Meng Gao, Kequan Xu, Xiangdong GongYe, Zhixiang Cheng, Ping Jiang, Xi Chen, Yufeng Yuan

**Affiliations:** 1 Department of Hepatobiliary and Pancreatic Surgery, Zhongnan Hospital of Wuhan University, Wuhan 430071, Hubei, PR China.; 2 Clinical Medicine Research Center for Minimally Invasive Procedure of Hepatobiliary & Pancreatic Diseases of Hubei Province, Wuhan 430071, Hubei, PR China.

**Keywords:** Hepatocellular carcinoma, CENPU, E2F6, E2F1, G1/S transition

## Abstract

Centromere protein U (CENPU), a centromere-binding protein required for cellular mitosis, has been reported to be closely associated with carcinogenesis in multiple malignancies; however, the role of CENPU in hepatocellular carcinoma (HCC) is still unclear. Herein, we investigated its biological role and molecular mechanism in the development of HCC. High CENPU expression in HCC tissue was observed and correlated positively with a poor prognosis in HCC patients. CENPU knockdown inhibited the proliferation, metastasis, and G1/S transition of HCC cells* in vivo* and* in vitro*, while ectopic expression of CENPU exerted the opposite effects. Mechanistically, CENPU physically interacted with E2F6 and promoted its ubiquitin-mediated degradation, thus affecting the transcription level of E2F1 and further accelerating the G1/S transition to promote HCC cell proliferation. E2F1 directly binds to the CENPU promoter and increases the transcription of CENPU, thereby forming a positive regulatory loop. Collectively, our findings indicate a crucial role for CENPU in E2F1-mediated signalling for cell cycle progression and reveal a role for CENPU as a predictive biomarker and therapeutic target for HCC patients.

## Introduction

Hepatocellular carcinoma (HCC), one of the most common malignancies, is the third major cause of tumour-related mortality 1, 2. Each year, there are approximately 700,000 new cases and 800 000 related fatalities 3. Despite a variety of prevention and therapeutic efforts, such as surgical excision, radiofrequency ablation, liver transplantation and molecularly targeted therapy, HCC patients have a five-year survival rate of less than 20% due to the absence of identifiable clinical symptoms in the early stages of HCC 3-5. Hence, a thorough grasp of the molecular basis and biological processes underlying HCC as well as the development of novel diagnostic and therapeutic targets are key components of HCC research.

The centromere protein (CENP) family is highly conserved, and its members function as attachment sites for spindle microtubules to promote chromosomal segregation during mitosis 6, 7. The family mainly includes CENPA, CENPB, CENPC, CENPM, CENPE, CENPT, CENPH, and CENPU 8. Importantly, CENPs have been demonstrated to have an essential role in carcinogenesis and tumour progression, except for their impact on mitosis. For example, silencing CENPA can reduce the aggressive phenotype of lung adenocarcinoma cells, and CENPA expression is tightly correlated with a poor prognosis in patients with lung adenocarcinoma 9, 10. CENP-H is markedly upregulated in colorectal cancers, and overexpression of CENPH was found to induce chromosome missegregation and aneuploidy in a diploid cell line 11. Moreover, downregulation of CENP-E leads to a prolonged cell cycle delay in cellular mitosis, and an inhibitor of CENPE has been reported to be a promising anticancer compound in sarcoma 12. Notably, our laboratory previously found that CENPM, another homologue of CENPs, could promote cell cycle progression and act as an antiapoptotic factor via the P53 signalling pathway in HCC 13.

Centromere protein U (CENPU), also known as polo-box-interacting protein 1 (PBIP1), interphase centromere complex protein 24 (ICEN24), or Cenp-50, is a centromere-binding protein that serves an important function in cellular mitosis and cell cycle progression 14-17. In human cells, CENPU deficiency can result in chromosomal attachment defects during mitosis 8. CENPU was also identified as a key mediator required for kinetochore-microtubule attachment via its interaction with Hec1 18. Moreover, as a phosphorylation substrate of Polo-like kinase 1 (Plk1), phosphorylated CENPU interacts with PLK1, recruiting PLK1 to interphase and mitosis kinetochores, which are crucial for proper mitotic progression 19. In addition to effects on kinetochore assembly, chromosome segregation, and mitosis progression, accumulating evidence indicates that CENPU dysregulation is associated with tumorigenesis. In breast cancer, CENPU has been reported to promote tumour development by activating the PI3K/AKT/NF-κB signalling pathway 20, 21. Another report demonstrated that CENPU knockdown could inhibit the growth and block the cell cycle process of lung cancer cells 22. Moreover, elevated CENPU expression could enhance the proliferation, invasion, and migration of ovarian cancer cells 23. A more recent study showed aberrantly high expression of CENPU in HCC, which was closely associated with the pathological stage 24. Nevertheless, the exact functions and molecular mechanisms of CENPU in the carcinogenesis and development of HCC have not yet been clarified.

In this research, we discovered that high expression of CENPU was significantly linked with a poor prognosis in HCC patients. Additionally, CENPU promoted hepatoma cell proliferation, invasion, migration, and cell cycle progression. Mechanistically, CENPU interacted with E2F6 and impaired its protein stability, thus eliminating the transcriptional repression on E2F1 and promoting HCC progression. Our results demonstrated that CENPU functions as an oncogene and might serve as a promising diagnostic and therapeutic target for HCC.

## Materials and Methods

### Patients and clinical specimens

Clinical samples were collected from 80 HCC patients who underwent hepatectomy at Zhongnan Hospital, Wuhan University (Hubei Province, China). All specimens were collected within 30 minutes immediately after hepatectomy. Subsequently, they were stored in liquid nitrogen with RNAlater (Catalogue Number AM7021, Invitrogen, USA). All patients were verified to have HCC by histological analysis following hepatectomy. In addition, informed consent was obtained from all patients prior to tissue collection and usage in the study. This study was approved by the ethical review boards of Zhongnan Hospital (KELUN2020100).

### Cell culture

Human hepatoma cell lines comprising SK-hep1, HepG2, Hep3B, Huh-7, HCCLM3, HCCLM6, MHCC-97H, MHCC-97L and PLC/PRF/5 and the immortalized human liver cell line HL-7702 (L02) were purchased from the Cell Bank of Type Culture Collection (CBTCC, Shanghai, China). All cells were grown at 37 °C in Dulbecco's modified Eagle's medium (DMEM)/high glucose (Gibco, USA) with 10% foetal bovine serum (Gibco, USA) in a humidified incubator containing 5% CO2.

### RNA extraction, reverse transcription, and quantitative real-time PCR

Total RNA was prepared from tissues and cells by utilizing TRIzol reagent (Catalogue Number 15596026, Invitrogen, USA). HiScript Ⅱ Q RT SuperMix (Catalogue Number R223-01, Vazyme, China) was used to reverse-transcribe cDNA into mRNA. qPCR was performed using Taq Pro Universal SYBR qPCR Master Mix (Catalogue Number Q712-02, Vazyme, China) on a CFX96TM Real-Time system (Bio-Rad, USA). All experiments were repeated three times, and the primer sequences are shown in Supplementary Table 1.

### RNA interference, plasmid construction, lentivirus construction, and cell transfection

Small interfering RNAs (siRNAs) targeting CENPU, E2F1, and E2F6 were designed and purchased from GeneCreate (Wuhan, China). The coding sequences of CENPU and E2F6 were separately cloned into the pcDNA3.1 vector. For plasmid and siRNA transfection, Lipofectamine 3000 (Catalogue Number L3000015, Invitrogen, USA) and GenMute (Catalogue Number SL100568, SignaGen, USA) were employed, respectively. GeneCreate (Wuhan, China) provided lentiviral small hairpin RNA (shRNA) targeting CENPU and E2F6, which was effectively transfected into the HCC cell lines. The siRNA and shRNA sequences are shown in Supplementary Table 1.

### Western blotting

SDS (1×) was utilized to extract protein samples. The concentration of the samples was calculated via a bicinchoninic acid assay, and equal amounts of protein were used for subsequent steps. Samples were subjected to sodium dodecyl sulphate-polyacrylamide gel electrophoresis and then transferred to PVDF membranes, which were blocked with 5% defatted milk for 1 h and incubated with the corresponding primary antibodies at 4 °C overnight. The next day, the membranes were incubated with secondary antibodies at room temperature for 1 h after being rinsed in TBST for 20 minutes. An ECL chemiluminescence imaging system (Tanon-5200, Shanghai, China) was used to detect the protein signals. The antibodies used in the current study are listed in Supplementary Table 2.

### Statistical analysis

SPSS v. 24.0 (IBM Corp, Armonk, NY, USA) was used to perform statistical analyses. Data from at least three independent experiments are presented as the mean ± standard deviation (MEAN+SD). The Kaplan-Meier method was used to assess the overall survival (OS) and recurrence-free survival (RFS) of HCC patients. The Student's t test and Spearman's correlation analysis were performed to analyse the data. *P* values are indicated as follows: *P < 0.05; **P < 0.01; ***P < 0.001.

The detailed procedures used for the other assays are described in the Supplementary Materials and Methods.

## Results

### CENPU was markedly upregulated in HCC and correlated with a poor prognosis

**Figure 1:**
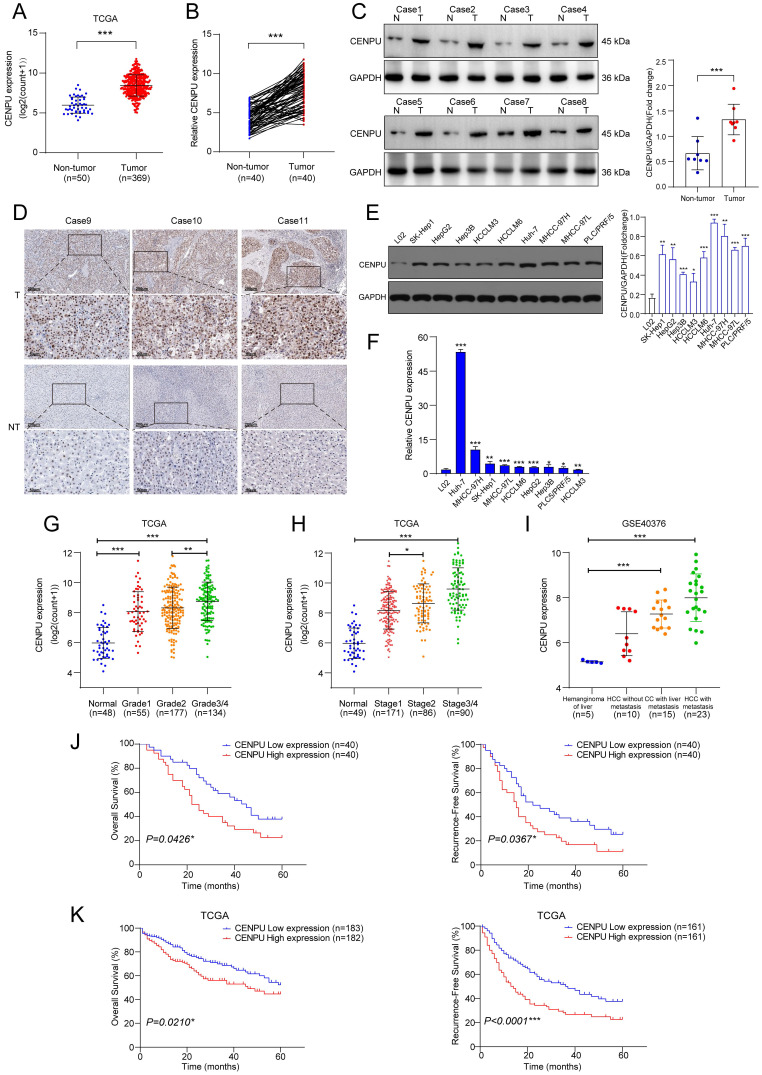
** CENPU was significantly elevated in HCC and related to poor prognostication. (A)** CENPU mRNA expression based on the TCGA-LIHC dataset. **(B)** qRT-PCR analyses of CENPU in 80 pairs of HCC samples. **(C-D)** CENPU protein expression in HCC clinical specimens was detected by immunoblotting and IHC analysis. **(E-F)** CENPU expression in hepatoma cell lines and immortalized human hepatocytes. **(G-H)** CENPU mRNA expression was correlated with HCC tumour grade and stage. **(I)** CENPU levels in metastatic and nonmetastatic HCC tissues based on GSE40467. **(J)** Kaplan-Meier analysis of OS and RFS in 80 HCC patients from Zhongnan Hospital. **(K)** Kaplan-Meier analysis of OS and RFS in 365 HCC patients according to the TCGA dataset. *p < 0.05; **p < 0.01; ***p < 0.001. T: tumour; NT: nontumor.

We first investigated the CENPU transcript level in multiple tumour types and found that CENPU expression was increased in the tumour group in comparison to the corresponding nontumor group for the majority of tumour types (Fig. S1). Analysis of the TCGA-LIHC dataset revealed that CENPU was markedly overexpressed in HCC (Fig. 1A). CENPU mRNA levels were also elevated in HCC specimens, according to qRT-PCR analysis of 80 pairs of HCC tumour and peritumour tissues (Fig. 1B). Next, western blotting and IHC validated the upregulated CENPU protein expression in HCC tissues (Fig. 1C, D). Likewise, hepatoma cell lines had higher CENPU expression than the normal hepatocyte line L02 at both the protein and mRNA levels (Fig. 1E, F). Moreover, clinical data analysis showed that a high level of CENPU expression was linked to tumour grade, stage, portal vein tumour thrombus (PVTT), and Barcelona Clinic Liver Cancer (BCLC) stage (Fig. 1G, H; Table 1). In addition, analysis of transcriptome datasets of GSE40367 demonstrated that CENPU expression increased in HCC patients with metastasis (Fig. 1I). Furthermore, 80 HCC patients were classified into the CENPU-Low and CENPU-High groups according to the median CENPU mRNA expression level. We found that the overall survival and relapse-free survival were shorter in patients with high CENPU expression via Kaplan-Meier survival analysis (Fig. 1J). Considering the limited number of patients involved in our study, we investigated the correlation between the CENPU expression level and the prognosis of HCC patients based on the TCGA-LIHC database. Consistently, Kaplan-Meier survival analysis indicated that high expression levels of CENPU were closely associated with poor overall survival and relapse-free survival (Fig. 1K).

### CENPU promoted the proliferation, invasion, and migration of HCC cells *in vitro*

**Figure 2:**
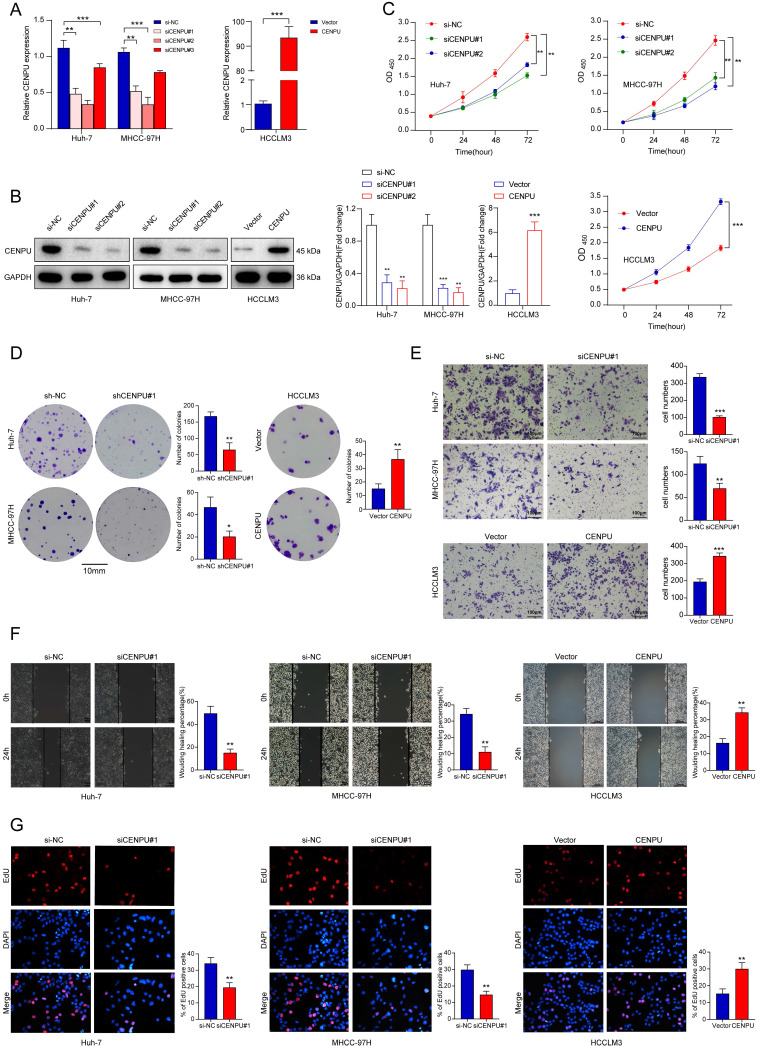
** CENPU enhanced the growth and migration of hepatoma cells. (A-B)** The efficiency of CENPU knockdown and overexpression was validated via qRT-PCR and immunoblotting. **(C-D)** The proliferative ability of Huh-7, MHCC-97H, and HCCLM3 cells was detected by CCK-8 and colony formation assays. **(E-F)** CENPU knockdown repressed the invasion and migration ability of Huh-7 and MHCC-97H cells, while CENPU overexpression reversed these results in HCCLM3 cells. **(G)** EdU assays indicating that CENPU silencing delayed the G1/S transition. *p < 0.05; **p < 0.01; ***p < 0.001.

To determine the biological function of CENPU in HCC, we chose Huh-7 and MHCC-97H as the knockdown models and HCCLM3 as an overexpression model based on the expression tendency in HCC cell lines. siRNA and plasmids were designed and transfected into cells. Forty-eight hours later, the transfection efficiency was monitored via qRT-PCR and immunoblotting (Fig. 2A, B). CCK8 and clonogenic assays were carried out to assess the proliferative capacity of HCC cells. The results showed that downregulation of CENPU repressed the proliferation of HCC cells, while overexpression of CENPU promoted proliferation (Fig. 2C, D; Fig. S2A). Moreover, Transwell and wound healing assays demonstrated that CENPU knockdown suppressed the invasion and migration of Huh-7 and MHCC-97H cells, whereas the reverse results were observed after CENPU overexpression in HCCLM3 cells (Fig. 2E, F; Fig. S2B, C). Additionally, considering that rapid cell cycle progression could lead to cancer proliferation, an EdU incorporation assay was conducted to examine the ratio of cells entering S phase. The results indicated that CENPU knockdown cells entered S phase at a lower rate than the control group, while CENPU-overexpressing cells entered S phase at a higher rate (Fig. 2G; Fig. S2D).

### Knockdown of CENPU inhibited the G1/S transition of HCC cells via E2F1

**Figure 3:**
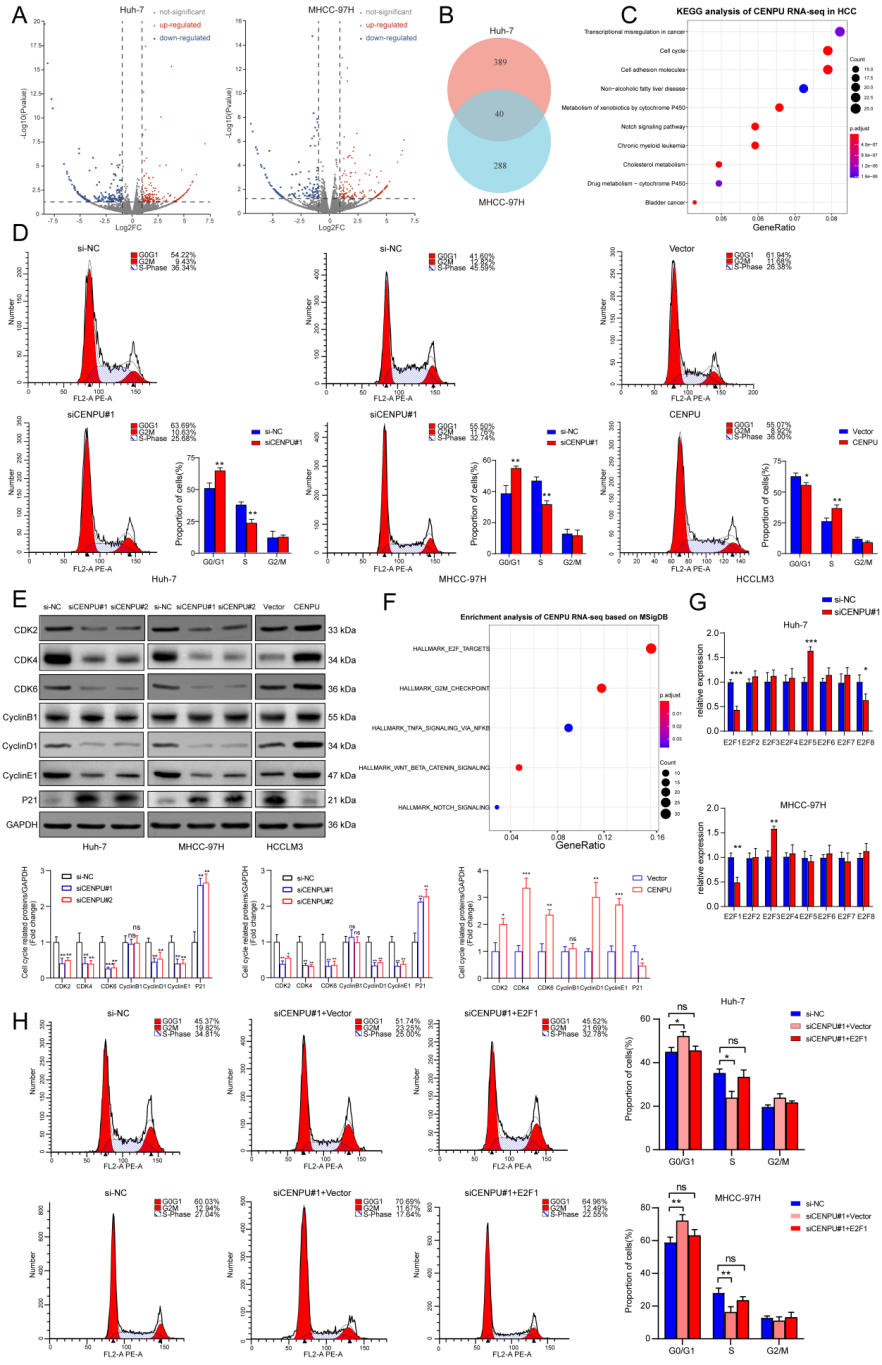
** CENPU promoted the G1/S transition of HCC cells through E2F1. (A-B)** Volcano plots and a Venn diagram were used to visualize the differentially expressed genes in Huh-7 and MHCC-97H cells transfected with si-CENPU or si-NC. **(C)** KEGG analysis of CENPU RNA-seq data in HCC cells. **(D)** The proportion of cells within divergent cell cycle stages was determined by flow cytometry analyses following CENPU knockdown or overexpression. **(E)** Western blotting of cell cycle-related proteins. **(F)** Enrichment analysis of CENPU RNA-seq data based on the Molecular Signatures Database. **(G)** qRT-PCR analysis of the E2F transcription factors in Huh-7 and MHCC-97H cells transfected with siRNA against CENPU. **(H)** Flow cytometry analyses indicated that E2F1 overexpression restored CENPU knockdown-mediated G1/S blockade. ns: no significance; *p < 0.05; **p < 0.01; ***p < 0.001.

To further investigate the oncogenic role of CENPU in HCC, we conducted RNA sequencing in Huh-7 and MHCC-97H cells after silencing CENPU (Fig. 3A, B). KEGG analysis of the differentially expressed genes in the two cell lines showed a notable enrichment of the cell cycle pathway, which was also consistent with the results of GO analysis and GSEA (Fig. 3C; Fig. S3A, B). Therefore, flow cytometry analyses were carried out to assess the influence of CENPU on the ratio of HCC cells in different cell cycle stages. The results demonstrated that downregulation of CENPU led to significant cell cycle arrest at G0/G1 phase, while overexpression of CENPU expedited the transition from G1 to S phase (Fig. 3D; Fig. S3C). Moreover, cell cycle-related proteins were detected by western blotting. The results indicated that CENPU knockdown induced marked downregulation of cyclin D1, cyclin E1, CDK2, CDK4, and CDK6, whereas the expression of P21 obviously increased and that of cyclin B1 remained the same. In contrast, CENPU overexpression resulted in the opposite changes (Fig. 3E). Taken together, these findings indicated that suppression of CENPU blocked the transition from G1 to S phase in hepatoma cells. Moreover, we found that the differentially expressed genes caused by CENPU knockdown were closely enriched in the E2F targets pathway based on the Molecular Signatures Database (MSigDB), consistent with the GSEA results (Fig. 3F; Fig. S3D). It is widely acknowledged that the E2F transcription factor family plays a vital role in cell cycle progression, especially in the G1/S transition, by transcriptional regulation of its target genes, such as cyclins D and E^25^-^27^. This led us to hypothesize that there might be some relationship between CENPU and the E2F family. Therefore, the mRNA expression of eight E2F transcription factors (TFs) in Huh-7 and MHCC-97H cells was detected by qPCR after knockdown of CENPU. As shown in Fig. 3G, only E2F1 expression simultaneously exhibited significant changes in Huh-7 and MHCC-97H cells. The results of the western blot analysis also revealed that knockdown of CENPU repressed E2F1 expression (Fig. S3E). As an important initiator for cells to enter S phase, E2F1 is engaged in the tumorigenesis and development of multiple malignancies 28. Next, flow cytometry analyses were conducted after CENPU siRNA and E2F1 vectors were cotransfected into HCC cells to determine whether E2F1 was involved in CENPU-mediated cell cycle progression. Transfection efficiency was ascertained with western blotting (Fig. S3F). As expected, ectopic E2F1 expression rescued the arrest of the G1/S transition induced by CENPU knockdown. In contrast, the acceleration of the G1/S transition caused by CENPU overexpression was reversed by silencing E2F1 (Fig. 3H; Fig. S3G). In conclusion, these results indicated that CENPU could regulate cell cycle progression by modulating E2F1 expression.

### CENPU promoted tumour growth and metastasis *in vivo*

**Figure 4:**
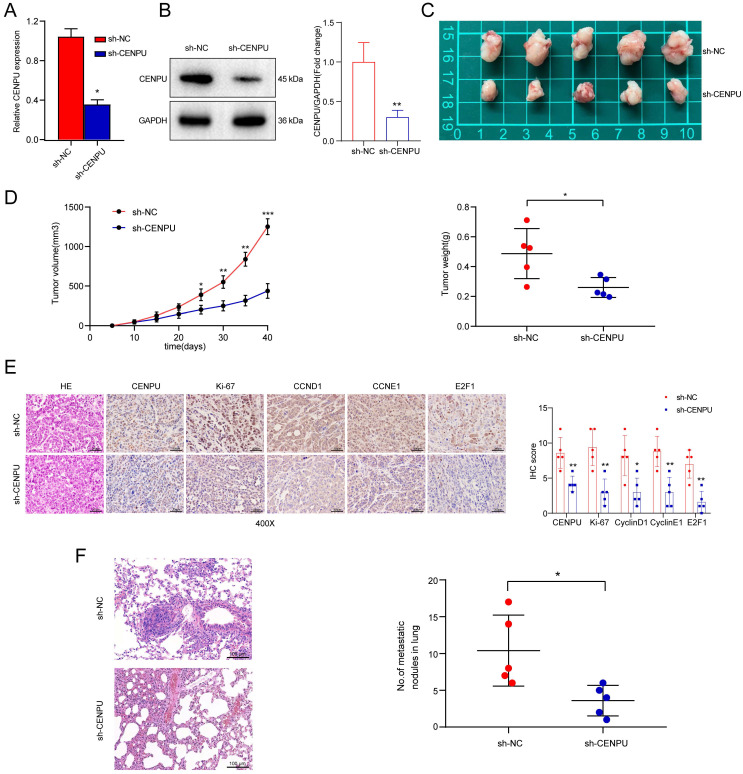
** CENPU knockdown repressed the formation and metastasis of xenograft tumours. (A-B)** Knockdown effectiveness of CENPU was confirmed by qRT-PCR and immunoblotting assays. **(C)** Representative images of tumours harvested from nude mice inoculated with stable CENPU knockdown (n=5) or negative control Huh-7 cells (n=5). **(D)** Tumour volume and weight were assessed at the specified time points following subcutaneous implantation. **(E)** The expression of CENPU, E2F1, and cell cycle-related proteins in xenograft tumours was determined by IHC. **(F)** Representative HE-stained images of lung metastatic tumours obtained from nude mice injected with stable CENPU knockdown Huh-7 cells or control cells. *p < 0.05; ***p < 0.001.

To assess the biological function of CENPU in HCC tumorigenesis *in vivo*, Huh-7 cells with stably downregulated CENPU expression (sh-CENPU) or control cells (sh-NC) were implanted subcutaneously into 10 male BALB/c nude mice, which were randomly divided into two groups. The CENPU silencing efficiency in Huh-7 cells was confirmed by qPCR and immunoblotting (Fig. 4A, B). The volume and weight of xenograft tumours decreased visibly following stable silencing of CENPU (Fig. 4C, D). Additionally, IHC staining showed decreased expression of Ki-67, cyclin D1, cyclin E1, and E2F1 in sh-CENPU-treated Huh-7 xenograft tumours (Fig. 4E). Furthermore, a pulmonary metastasis model was constructed by injecting tumour cells intravenously. HE staining and quantitative microscopic analysis revealed that mice in the sh-CENPU group showed fewer lung metastasis nodules (Fig. 4F). In brief, CENPU enhanced the tumour formation, growth, and metastasis of HCC *in vivo*.

### E2F6 interacted with CENPU and downregulated E2F1 at the transcriptional level

**Figure 5:**
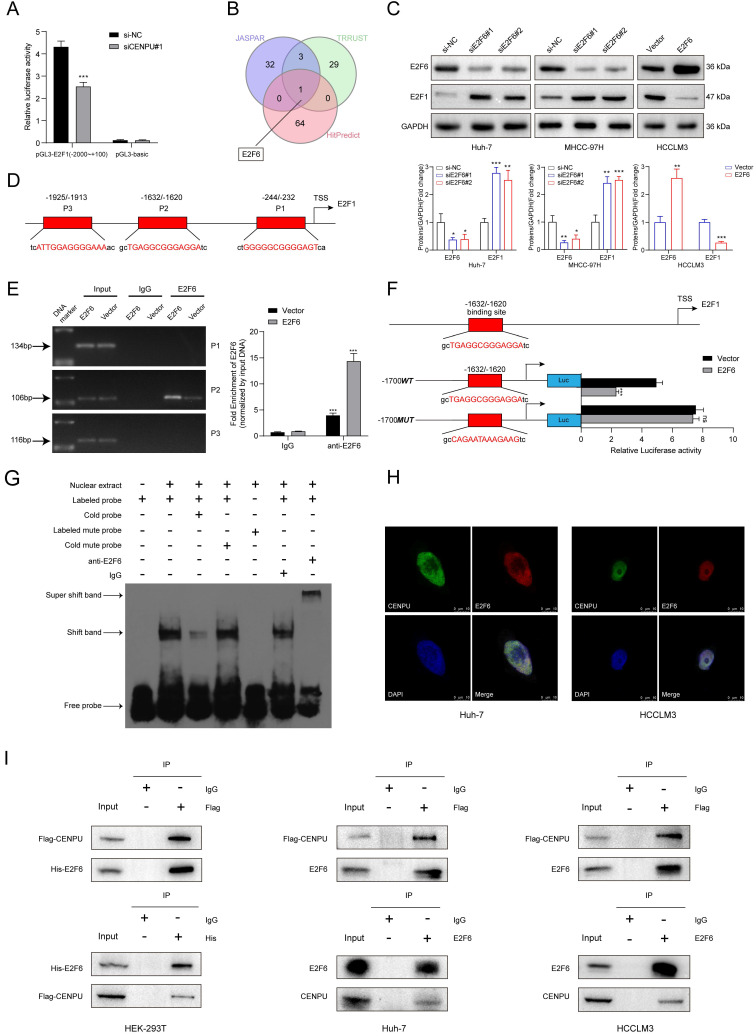
** E2F6 inhibited E2F1 transcription and interacted with CENPU. (A)** Luciferase activity was measured after Huh-7 cells were cotransfected with the full-length fragment of the E2F1 promoter (-2000~+100) and si-CENPU. **(B)** Venn diagram analysis of predicted proteins that could simultaneously interact with CENPU and regulate E2F1 transcription. **(C)** Western blotting analysis of E2F1 following E2F6 knockdown or overexpression in HCC cells. **(D)** Schematic representation of the E2F6 binding site on the E2F1 promoter region. **(E)** ChIP-PCR and ChIP-qPCR results showing E2F6 binding to the E2F1 promoter at the -1632/-1620 site in Huh-7 cells. **(F)** Luciferase activity was measured after Huh-7 cells were cotransfected with the E2F1 promoter fragment (WT or MUT) and E2F6-overexpression plasmid. **(G)** EMSA was conducted to validate the binding of E2F6 to the E2F1 promoter sequences. **(H)** Typical IF imaging of the colocalization of CENPU and E2F6 in Huh-7 and HCCLM3 cells. **(I)** Exogenous and endogenous co-IP assays confirmed the interaction between CENPU and E2F6. ns: no significance; ***p < 0.001.

We further explored the underlying mechanism by which CENPU regulates E2F1 expression. Given that E2F1 mRNA expression was downregulated by CENPU knockdown, we wondered whether CENPU could regulate the transcriptional activity of the E2F1 promoter or affect E2F1 mRNA stability. The results of the dual-luciferase activity and RNA stability assays showed that after silencing CENPU, the luciferase activity of the E2F1 promoter region was significantly decreased, while the half-life of E2F1 mRNA was unaffected compared with the control group (Fig. 5A; Fig. S4A). Since CENPU lacks a recognizable DNA-binding domain and cannot act as a transcription factor itself, we speculated that CENPU might regulate the transcriptional activity of E2F1 via other TFs. Therefore, potential transcription factors of E2F1 that could interact with CENPU were predicted using the HitPredict, JASPAR, and TRRUST websites. One candidate (E2F6) was obtained through Venn diagram analysis (Fig. 5B; Table S5). We next examined whether E2F6 acted as a transcription factor of E2F1. E2F1 expression was markedly decreased after E2F6 overexpression but enhanced after E2F1 knockdown at both the mRNA and protein levels (Fig. 5C; Fig. S4B, C). We further investigated whether E2F6 could directly regulate E2F1, and three probable binding loci in the E2F1 promoters were discovered through the JASPAR database (Fig. 5D; Fig. S4D). The results of the ChIP-PCR analysis showed that E2F6 interacted with the E2F1 promoter at the -1632/-1620 sites (Fig. 5E). Moreover, a dual-luciferase activity assay showed that E2F1 transcriptional activity was significantly inhibited when Huh-7 cells were transfected with the E2F6 overexpression plasmid and was not affected when we mutated the sequences of the -1632/-1620 E2F1 promoter (Fig. 5F). Additionally, an EMSA was performed to further confirm our hypothesis. The results indicated that the labelled probe, including the -1632/-1620 sites of the E2F1 promoter sequence, could specifically bind with nuclear protein extracts of Huh-7 cells (Fig. 5G).

To determine whether E2F6 interacted with CENPU, confocal immunofluorescence staining was conducted, and the results showed that E2F6 was mainly colocalized in the nucleus with CENPU in Huh-7 and HCCLM3 cells (Fig. 5H). Then, exogenous and endogenous co-IP assays were performed to confirm the interplay. HEK-293T cells were cotransfected with Flag-EGFP-tagged CENPU and His-Myc-mCherry-tagged E2F6 and then subjected to reciprocal co-IP assays. Western blotting revealed that Flag-CENPU interacted with His-E2F6 (Fig. 5I). Importantly, the interaction between CENPU and E2F6 at the endogenous protein level was observed in both HCCLM3 and Huh-7 cells transfected with Flag-EGFP-tagged CENPU (Fig. 5I). Taken together, the above results indicated that CENPU interacted with E2F6, which directly bound to the promoter region of E2F1.

### CENPU repressed E2F6 protein stability via the ubiquitin-proteasome pathway

**Figure 6:**
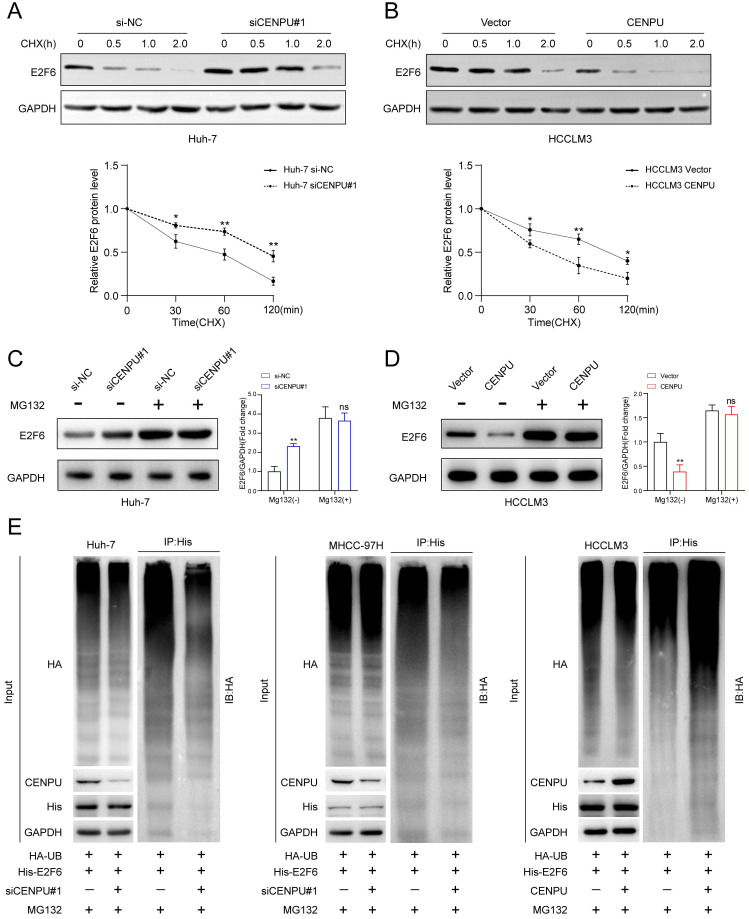
** CENPU promoted the proteasomal degradation of E2F6. (A-B)** Immunoblotting analysis of E2F6 in Huh-7 and HCCLM3 cells after treatment with CHX for 0 h, 0.5 h, 1 h, and 2 h. **(C-D)** MG132 (10 µM) was applied to Huh-7 and HCCLM3 cells for 4 h and then immunoblotted for E2F6. **(E)** MG132 (20 µg/ml) was applied to HCC cells transfected with His-E2F6, HA-ubiquitin, CENPU plasmid, or si-CENPU, and 4 h later, total protein was extracted and subjected to co-IP using anti-His antibody.

Considering that CENPU interacted with E2F6, we wondered whether this interaction could influence the expression of E2F6. qRT-PCR and immunoblotting were performed after CENPU silencing or overexpression. Interestingly, only the protein expression level but not the mRNA expression level of E2F6 was affected after CENPU knockdown or overexpression (Fig. S5A, B). We subsequently used IHC analysis to detect the protein expression of E2F6 in clinical samples. Notably, E2F6 protein expression was markedly increased in HCC tissues with reduced CENPU expression levels, while Spearman's correlation analysis showed that such an inverse association did not exist between CENPU and E2F6 at the mRNA level (Fig. S5C-E). This result led us to hypothesize that CENPU regulated E2F6 protein stability. Then, cycloheximide (CHX) chase assays were conducted, and the results indicated that the half-life of E2F6 was prolonged in CENPU knockdown cells, whereas the half-life of E2F6 was shortened in CENPU-overexpressing cells (Fig. 6A, B). It is well acknowledged that the ubiquitin-proteasome degradation pathway plays an essential role in regulating intracellular protein levels. Importantly, the degradation of E2F6 caused by CENPU could be antagonized by the proteasome inhibitor MG132 (Fig. 6C, D). Therefore, a ubiquitination assay was conducted, and we found that CENPU downregulation was accompanied by a decrease in ubiquitinated E2F6 in Huh-7 and MHCC-97H cells, while CENPU overexpression was accompanied by enhanced ubiquitination of E2F6 in HCCLM3 cells (Fig. 6E). In conclusion, CENPU physically interacted with E2F6 and impaired E2F6 protein stability through the ubiquitin-proteasome pathway.

### E2F6 was crucial for CENPU-regulated tumour progression in HCC cells

**Figure 7:**
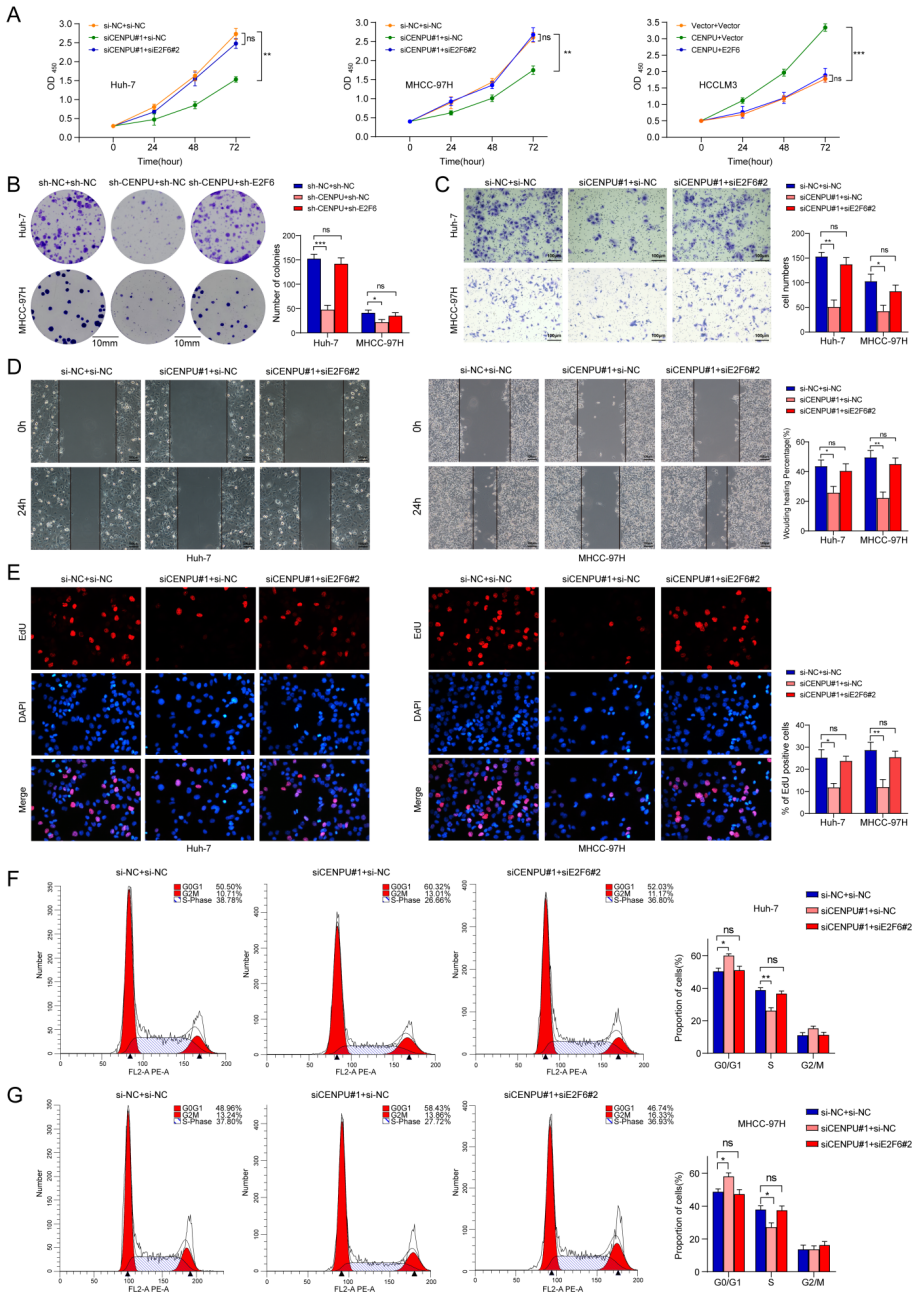
** E2F6 was required for CENPU-mediated HCC cell growth, invasion, migration, and cell cycle progression. (A-B)** CCK-8 and clonogenic assays of Huh-7 and MHCC-97H cells after simultaneous knockdown of E2F6 and CENPU. **(C-D)** Transwell and wound healing assays showed that silencing E2F6 nullified the CENPU knockdown-mediated inhibitory effects on cell invasion and migration. **(E-F)** EdU and flow cytometry analyses were carried out to evaluate the impact of E2F6 downregulation on the distribution of cell cycle phases. ns: no significance; *p < 0.05; **p < 0.01.

To further explore whether E2F6 was required for the regulatory effect of CENPU on HCC cell proliferative capacity and cell cycle processes, we conducted rescue experiments after simultaneously silencing CENPU and E2F6 in HCC cells. CCK-8 and clonogenic assays showed that the repression of cell proliferative capacity caused by CENPU knockdown in HCC cells was ameliorated after silencing E2F6 (Fig. 7A, B). Consistently, CENPU overexpression enhanced cell viability, which was reversed by the upregulation of E2F6 (Fig. S6A). Additionally, Transwell and wound healing assays demonstrated that the suppression of invasion and migration mediated by CENPU knockdown were counteracted when E2F6 was downregulated (Fig. 7C, D; Fig. S6B, C). In line with the abovementioned results, the EdU assay and flow cytometry analyses showed that knockdown of E2F6, but not the negative control, partially counteracted the G1/S transition block caused by CENPU silencing (Fig. 7E, F, G). Conversely, E2F6 upregulation partially delayed the accelerated G1/S transition due to CENPU elevation (Fig. S6D, E). Based on these findings, we propose that E2F6 is required for CENPU-mediated proliferation, migration, and cell cycle progression of hepatoma cells.

### E2F1 promoted CENPU expression transcriptionally to form a positive feedback loop

**Figure 8:**
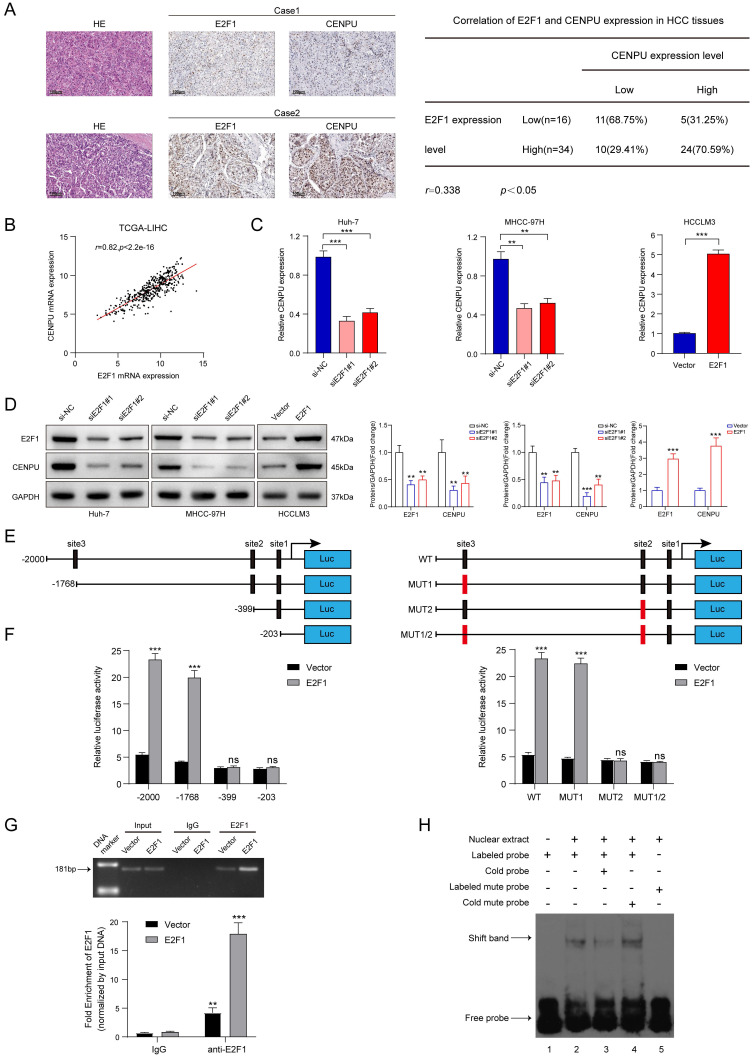
** E2F1 could target the CENPU promoter to increase its expression. (A)** Representative images of IHC staining of E2F1 and CENPU in HCC tissues (n=50). High E2F1 expression in patients were accompanied with elevated CENPU expression. **(B)** The correlation between E2F1 and CENPU mRNA expression was determined based on the TCGA-LIHC dataset (n=369) (Spearman's correlation analysis). **(C-D)** qRT-PCR and immunoblotting analysis of CENPU following E2F1 knockdown or overexpression in HCC cells. **(E)** Schematic illustration of luciferase reporter vectors containing different fragments and mutants of the CENPU promoter region. **(F)** Dual-luciferase reporter assays were performed in Huh-7 cells to confirm the exact E2F1 binding region on the CENPU promoter. **(G)** ChIP-qPCR and ChIP-PCR analysis of E2F1 binding to the CENPU promoter. **(H)** The protein-DNA interaction between the E2F1 and CENPU promoter sequences was assessed via EMSA. ns: no significance; **p < 0.01; ***p < 0.001.

We next screened for the upstream mediator of CENPU in HCC. Interestingly, E2F1 was predicted to possess the potential to bind to the CENPU promoter using the UCSC databases (Table S6). First, the expression profiles of E2F1 were obtained via bioinformatics analytics based on the TCGA-LIHC dataset. The results showed that E2F1 transcript levels were dramatically enhanced in HCC tissues compared with normal tissues (Fig. S7A). Consistently, qRT-PCR analysis of 80 paired HCC patient tissues indicated that tumour tissues had a higher level of E2F1 expression than peritumour tissues (Fig. S7B). Additionally, Kaplan-Meier survival analysis suggested that elevated E2F1 expression was evidently linked to poor prognostication in HCC patients (Fig. S7C). Then, the biological function of E2F1 in HCC was briefly verified. We found that knockdown of E2F1 inhibited the proliferation ability and G1/S transition of hepatoma cells, whereas overexpression of E2F1 produced the opposite effects (Fig. S7D-H). Importantly, IHC staining revealed that CENPU expression levels were higher in HCC patients with elevated E2F1 expression (Fig. 8A). Spearman's correlation analysis suggested a positive correlation between E2F1 and CENPU mRNA expression (Fig. 8B). In addition, qRT-PCR and immunoblotting analysis showed that CENPU expression was downregulated after E2F1 silencing but elevated after E2F1 overexpression (Fig. 8C, D). Next, a luciferase reporter assay was implemented in Huh-7 cells to further confirm whether E2F1 could act as an upstream regulator of CENPU. A series of truncations and mutants of the CENPU promoter were constructed according to the E2F1 binding motif and three potential binding sequences in the CENPU promoter (Fig. 8E; S7I, J). The results indicated that the enhancement of luciferase activity in the E2F1-overexpressing group was distinctly counterbalanced when site 2 (-410/-400) was deleted or mutated. However, this phenomenon was not observed when the other sites were altered, which suggested that site 2 was the binding region of E2F1 on the CENPU promoter (Fig. 8F). In addition, the ChIP and EMSA results further confirmed that E2F1 could bind directly to the -410/-400 sites in the CENPU promoter region (Fig. 8G, H). Collectively, these results indicated that E2F1 bound to the promoter region of CENPU to increase its expression; thus, E2F1/CENPU/E2F6 could form a positive feedback loop in HCC.

## Discussion

Despite far-reaching advancements in HCC diagnosis and therapeutic schemes, the morbidity and mortality rates of HCC patients remain high due to a limited understanding of the underlying process of tumour development and progression. Thus, there is an urgent need to understand the regulatory signalling pathway implicated in HCC and identify new potential biomarkers used for molecular-targeted treatment. CENPU, a constitutive protein of the centromere-associated network, plays a vital role in kinetochore-microtubule attachment, sister chromatid adhesion, and cell cycle progression 29, 30. Accumulating evidence shows that the expression of CENPU is dysregulated in several malignancies. However, the involvement of CENPU in carcinogenesis and its clinical relevance remain poorly explored in HCC. In this study, for the first time, we discovered that CENPU expression was markedly elevated in HCC, which was considerably correlated with a poor prognosis in HCC patients. Cellular phenotypic experiments indicated that CENPU promoted the proliferation, metastasis, and cell cycle progression of hepatoma cells *in vivo* and *in vitro*. Moreover, CENPU interacted with E2F6 and impaired its protein stability, thus eliminating the transcriptional repression on E2F1 and accelerating the G1/S transition. Collectively, our findings shed light on the role of CENPU in the tumorigenesis and progression of HCC.

The E2F family of transcription factors is a crucial group of modulators that exert an essential impact on cell cycle control, proliferation, apoptosis, DNA replication, and repair by regulating their target genes 31-33. The family consists of eight members, E2F1-E2F8, which can be further divided into activators (E2F1-E2F3a), repressors (E2F3b-E2F5), and inhibitors (E2F6-E2F8) according to their functional properties and structural characteristics 34, 35. As the most thoroughly investigated member of the E2F family, E2F1 plays an essential role in various malignancies 36. However, E2F1 appears to serve both as a tumour repressor and an oncogene in HCC. On the one hand, E2F1 acts as the transcription factor of p53 to augment its expression, inhibiting tumour growth in HCC 37, 38. On the other hand, E2F1 exhibits antiapoptotic activity in human and rodent hepatoma via its capacity to offset c-Myc-induced apoptosis 39. Moreover, E2F1 boosts the proliferative capacity of hepatoma cells by forming a positive regulatory loop with USP11 40. Additionally, E2F1-mediated DDX11 transcriptional stimulation can activate the PI3K/AKT/mTOR signalling pathway, culminating in enhanced migration and invasion capability of HCC cells 41. In the current study, we found that the abnormal upregulation of E2F1 was consistent with the malignant phenotype of HCC cells. Importantly, E2F1 overexpression promoted the G1/S transition, while E2F1 knockdown blocked the G1/S transition. It is well acknowledged that misregulation of the G1/S transition leads to uncontrolled cell proliferation and consequently promotes tumour development. Based on our findings, E2F1 might behave as an oncogene in HCC progression.

Given the roles played by E2F1 in the cell cycle and tumorigenesis, it is vital to determine the underlying mechanisms of E2F1 regulation. The most extensively studied CDK/RB/E2F axis is the primary mode of E2F1 regulation 42, 43. Moreover, transcriptional control, subcellular localization, and posttranslational modifications further complicate the regulatory network 44. Transcriptionally, E2Fs are largely self-regulated 44. One previous study demonstrated that E2F6, a pRb-independent transcriptional repressor, can suppress E2F1 transcriptionally in HEK293T cells 45. Consistently, in the current study, we first discovered that E2F6 could bind to the promoter of E2F1 and thus inhibit its expression in HCC cells. In addition, Pei et al. reported that E2F1 suppresses the interaction between E2F6 and EBNA3C to weaken the protein stability of E2F6, which is recruited by EBNA3C to bind the E2F1 promoter and inhibit its activity 46. Furthermore, a recent study revealed that E2F6 can be deubiquitinated and stabilized by USP22 and thereby inhibit the transcription of DUSP1, which in turn activates AKT signalling and leads to HCC progression 47. However, DUPS1 can also be regulated transcriptionally by E2F1 under oxidative stress. E2F1 binds directly to the promoter region of DUSP1 and activates its transcriptional activity, thus dephosphorylating MAP kinases, inducing apoptosis and suppressing tumour growth 48. The above findings indicate substantial functional redundancy and antagonism among E2Fs, adding more intricacy to E2F regulation. Thus, the coordinated network of E2Fs requires further investigation.

As a main pathway responsible for the degradation of 80% of intracellular proteins 49, the ubiquitin-proteasome system can regulate E2F protein expression at the posttranslational level 44. Several studies have clarified the effect of the ubiquitin-proteasome pathway on the function of E2F1 and consequent tumour development 40, 50, 51, while posttranslational modification of E2F6 in HCC cells is poorly understood. In the current study, we found that CENPU interacted with E2F6 and enhanced its ubiquitination, thereby impairing E2F6 protein stability. However, given that the lysosomal/autophagic pathway is also involved in the degradation of proteins, further experiments are needed to confirm the engagement of the lysosomal/autophagic pathway in E2F6 protein degradation. Moreover, it has been demonstrated that the deubiquitinating enzyme USP22 can directly interact with and stabilize E2F6 in HCC cells, which indicates the presence of corresponding E3 ubiquitin ligases of E2F6 forming a dynamic bidirectional regulatory system for ubiquitin modifications 47. We analysed and predicted a series of ubiquitylation loci and probable E3 ligases of E2F6 via Ubibrowser (Table S7); however, the precise molecular mechanism underlying the interaction between the E3 ubiquitin ligases/deubiquitinating enzymes, CENPU and E2F6 requires further exploration.

## Conclusion

**Figure 9:**
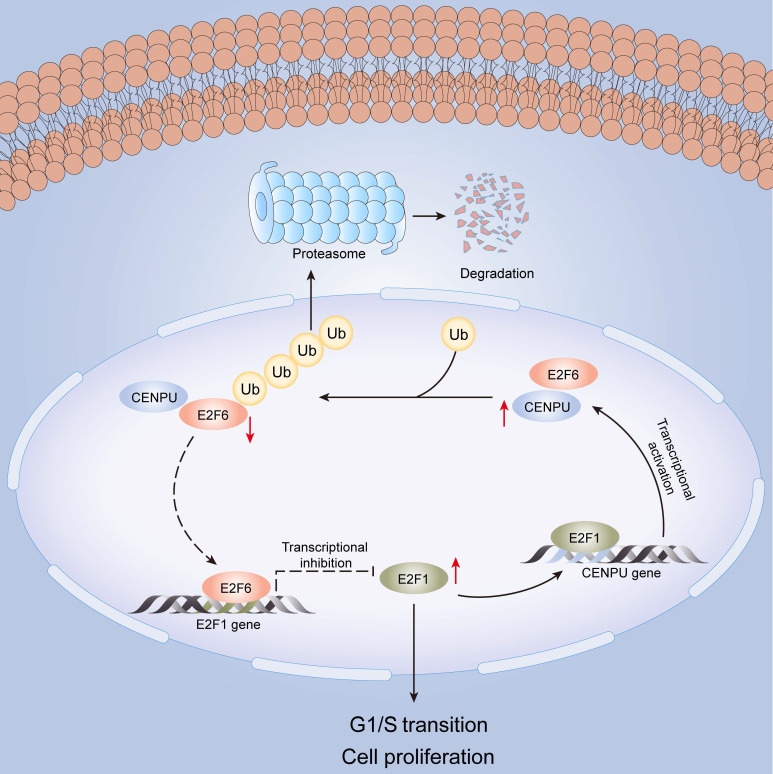
A model of the regulatory mechanisms of CENPU in HCC.

In this research, we identified for the first time that CENPU is an oncogene that promotes hepatoma cell proliferation, migration, and G1/S transition *in vitro* and *in vivo*. CENPU interacts with E2F6 and increases its ubiquitylation degree, thus affecting the expression of E2F1 transcriptionally and further altering cell proliferation and other phenotypes in HCC. Consequently, our findings unveil the role of CENPU in HCC carcinogenesis and development, establishing a theoretical basis for the development of potential prognostic biomarkers and novel therapeutic targets in HCC (Fig. 9).

## Supplementary Material

Supplementary methods, figures and tables.

## Figures and Tables

**Table 1:** Correlation between CENPU expression and clinicopathologic parameters in HCC patients

Characteristics	Patients	CENPU expression		*p* value
	N=80	High	Low	
**Age**				0.4912
< 65	49	23	26	
≥ 65	31	17	14	
**Gender**				0.5923
Female	18	10	8	
Male	62	30	32	
**Tumour Size (cm)**				0.1521
< 5	54	24	30	
≥ 5	26	16	10	
**AFP (μg/L)**				0.1709
< 400	48	21	27	
≥ 400	32	19	13	
**PVTT**				0.0253*
No	64	28	36	
Yes	16	12	4	
**TNM stage**				0.0147*
I+II	56	23	33	
III+IV	24	17	7	
**Cirrhosis**				0.4693
No	25	11	14	
Yes	55	29	26	
**HBV infection**				0.1035
No	29	11	18	
Yes	51	29	22	
**Lymph metastasis**				0.7094
No	72	37	35	
Yes	8	3	5	
**BCLC stage**				0.0452*
Low	58	25	33	
High	22	15	7	

PVTT: portal vein tumour thrombus; BCLC: Barcelona Clinic Liver Cancer. Bold italics indicate statistically significant values (*p < 0.05).
