# A positive feedback loop of CENPU/E2F6/E2F1 facilitates proliferation and metastasis via ubiquitination of E2F6 in hepatocellular carcinoma: Erratum

**DOI:** 10.7150/ijbs.85198

**Published:** 2023-06-21

**Authors:** Yingyi Liu, Ye Yao, Bo Liao, Hao Zhang, Zhangshuo Yang, Peng Xia, Xiang Jiang, Weijie Ma, Xiaoling Wu, Chengjie Mei, Ganggang Wang, Meng Gao, Kequan Xu, Xiangdong GongYe, Zhixiang Cheng, Ping Jiang, Xi Chen, Yufeng Yuan

**Affiliations:** 1 Department of Hepatobiliary and Pancreatic Surgery, Zhongnan Hospital of Wuhan University, Wuhan 430071, Hubei, PR China.; 2 Clinical Medicine Research Center for Minimally Invasive Procedure of Hepatobiliary & Pancreatic Diseases of Hubei Province, Wuhan 430071, Hubei, PR China.

After the publication of our paper, we noticed an error in Fig. 2G. Specifically, a panel of representative images were misplaced into the EdU results (si-NC group of Huh-7 cells) during the process of our final figure assembly. We carefully verified the original data again, corrected the mis-incorporated images and provided the correct version of Figure 2G below.

The conclusion of our research was not affected by the error. In this respect, all authors have agreed to the erratum, and we sincerely regret any annoyance the negligence in our work may have caused.

## Figures and Tables

**Figure 2:**
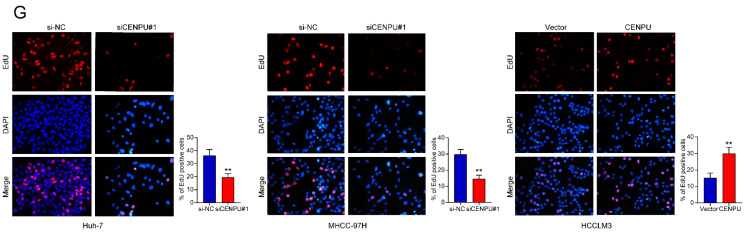
Corrected figure for original Figure 2G.

