# Supplementary material for: A positive feedback loop of CENPU/E2F6/E2F1 facilitates proliferation and metastasis via ubiquitination of E2F6 in hepatocellular carcinoma
Source: Int J Biol Sci. 2022 Jun 21;18(10):4071–87. doi: 10.7150/ijbs.69495 (PMC9274498; doi:10.7150/ijbs.69495)
Supplement: Supplementary file 1 — Supplementary methods, figures and tables. [file ijbsv18p4071s1.pdf]

Supplementary Materials and Methods

Immunohistochemistry (IHC)

Clinical specimens were fixed using 4% formalin, embedded in paraffin, and then cut into sections. The sections were deparaffinized and rehydrated in xylene and ethanol and then heated at 105°C in citrate buffer solution for 10 minutes for antigen repair. Next, the sections were treated with a 3% hydrogen peroxide solution for 10 minutes to inhibit endogenous peroxidase activity. After blocking with bovine serum albumin (BSA), the sections were incubated with primary antibodies at 4°C overnight and secondary antibodies for 1 h at room temperature. Finally, the sections were stained and dehydrated using haematoxylin, graded alcohol, and xylene. The percentage of positively stained cells was scored using five categories: 0 (< 5%), 1 (5–25%), 2 (26–50%), 3 (51–75%), and 4 (> 75%). The staining intensity was graded using four categories: 0 (negative), 1 (weakly positive), 2 (moderately positive), and 3 (strongly positive). The final staining index was obtained by multiplying the two scores. Samples with a final staining score of ≤4 were considered to exhibit low expression, and those with a score of >4 were considered to have high expression. Two independent investigators who were blinded to the patient characteristics scored the staining assessments.

Cell proliferation assays

The viability of cells was measured via the Cell Counting Kit-8 (Catalogue Number BS350A, Biosharp, China). Cells were plated into 96-well plates at a density of 5×10^3 cells per well. Then, 10 μl Cell Counting Kit-8 reagent was applied to the samples for 1 h. Finally, the absorbance was evaluated at 450 nm using a microplate reader.

Clonogenic assays

Clonogenic assays were conducted by seeding approximately 1000 cells into each well of 6-well plates for 2 weeks. Paraformaldehyde and 0.1% crystal violet solution were used to fix and stain the desired colonies. Subsequently, the colonies were counted under a microscope.

Cell migration and invasion assays

A wound healing assay was used to measure the cell migration ability. Approximately 10^6 cells were seeded into 6-well plates and cultured in DMEM without FBS for 24 h. Wounds were generated with a 100-μl plastic pipette tip. Then, images of the wounds were captured at 0 and 24 h. The areas of the scratches were calculated to assess the cell migration ability. The invasion ability of cells was measured using 24-well Transwell plates with a pore size of 8.0 μm. In the upper chamber covered with Matrigel, cells were seeded at a density of 2×10^4 per well in DMEM without serum. DMEM with 10% FBS was placed in the lower chamber as a stimulus to trigger cell invasion. After 24–72 h, the invaded cells were fixed with paraformaldehyde, stained with 0.1% crystal violet solution, and counted.

EdU incorporation assays

An EdU incorporation assay was performed using the EdU Staining Proliferation Kit (Catalogue Number ab222421, Abcam, UK) according to the manufacturer's protocol. Briefly, cells were incubated with culture medium containing 20 μM EdU reagent for 2 h at 37°C. The nuclei were stained with DAPI. Then, EdU-positive cells fixed with paraformaldehyde were

observed and counted under a fluorescence microscope (Olympus, Japan).

Cell cycle analysis

Cell cycle analysis was conducted utilizing a cell cycle staining kit (Catalogue Number CCS012, MultiSciences, China) in accordance with the manufacturer's instructions. The proportion of stained cells in G0/G1, S, and G2/M phase was detected with a CytoFLEX flow cytometer (Beckman, China). The data were analysed using FlowJo software (Tree Star, USA).

In vivo experiment

Four-week-old male BALB/c (nu/nu) mice were purchased from SPF Biotechnology (Beijing, China) and raised under specific pathogen-free (SPF) conditions. For xenograft experiments, Huh-7 cells (5×10^6) were subcutaneously implanted into the left axilla of BALB/c nude mice and stably transfected with sh-CENPU or sh-NC. Every 5 days, the tumour size was measured with a Vernier scale. Four weeks later, the mice were sacrificed, and the subcutaneous tumours were harvested for weighing and IHC analysis. For the lung metastasis assays, nude mice were inoculated with stably transfected cells (10^5 cells/mouse) through the tail vein. Five weeks later, the mice were euthanized, and the lungs were collected for haematoxylin-eosin (HE) staining.

RNA sequencing

Briefly, the concentration and integrity of total RNA was evaluated using a Qubit 2.0 Fluorometer (Life Technologies, USA) and Agilent 2100 Bioanalyzer (Agilent Technologies, USA). Then, the mRNA was enriched using magnetic beads with oligo(dT) and randomly

fragmented with cation (Mg2+). Using mRNA as a template, cDNAs were synthesized with six-base random hexamers, buffer, dNTPs, RNase H, and DNA polymerase I. Next, purified cDNAs were end-repaired and ligated to sequencing adapters. The screened cDNAs (~200 bp) were amplified by PCR to construct the mRNA-seq libraries and sequenced on the Illumina HiSeqTM 2000 system (Illumina, USA).

Dual-luciferase reporter activity assay

The binding of transcription factors and their target promoter regions was detected by a luciferase reporter assay. The sequences of wild-type (WT), mutant (MUT), and different lengths of the promoter regions of CENPU and E2F1 were inserted into the pGL3-basic vector, which was cotransfected with pcDNA3.1-E2F6 or pcDNA3.1-E2F1 into Huh-7 cells. Forty-eight hours following transfection, luciferase activity was measured using the Dual Luciferase® Reporter Assay System (Promega, Madison, USA).

RNA stability assays

To determine the stability of E2F1 mRNA, 5 μg/ml actinomycin D (Catalogue Number A4262, Sigma, USA) was applied to cells after transfection for 24 h. Total RNA was harvested at the indicated time points and subjected to qRT–PCR analysis.

Chromatin immunoprecipitation (ChIP)

ChIP assays were carried out using the SimpleChIP Enzymatic Chromatin IP Kit (Catalogue Number 9002S, Cell Signaling Technology, USA) in accordance with the manufacturer's

protocol. Briefly, fixed cells were lysed via ultrasound and then incubated with specific antibodies at 4°C overnight. DNA fragments were isolated from the DNA–protein complex by immunoprecipitation, elution, and decrosslinking. Finally, the purified DNA samples were subjected to PCR and qPCR analysis. The sequences of the primers used in the ChIP assay are presented in Supplementary Table 4.

Coimmunoprecipitation (co-IP)

Huh-7 and HCCLM3 cells transfected with Flag-EGFP-tagged CENPU were digested using trypsin-EDTA (Gibco, USA) and centrifuged. The cell precipitate was lysed in IP lysis buffer at 4°C for 1 h. Then, the cell lysate was incubated with 2 μl FLAG antibody or IgG overnight at 4°C. The next day, 20 μl magnetic beads were added and incubated with the protein-antibody complex for 3 h at 4°C. Subsequently, the beads were separated from the complex using a magnetic separation device and rinsed for 5 minutes with wash buffer. Finally, the proteins were separated after the bead-protein complex was heated with 2× SDS loading buffer at 96°C for 10 minutes. For exogenous co-IP, Flag-EGFP-tagged CENPU and His-Myc-mCherry-tagged E2F6 were transfected into HEK-293T cells, and the subsequent steps were identical to those used for the endogenous co-IP protocol.

Immunofluorescence (IF)

Triton X-100 (0.5%) was used to permeabilize the fixed cells with 4% paraformaldehyde. Then, the cells were incubated with primary antibody for 2 h at room temperature. Next, the cells were treated with fluorescein-conjugated secondary antibody for 1 h. Finally, a confocal

microscope (Zeiss LSM880) was used to detect and observe the fluorescence signal.

Electrophoretic mobility shift assay (EMSA)

An EMSA was performed to determine the binding of transcription factors and their targeted DNA sequences. Nuclear extract was prepared from Huh-7 cells using the NE-PER Nuclear and Cytoplasmic Extraction Kit (Catalogue Number 78833, Thermo, USA). The sequence of the double-stranded probe used for E2F1 binding was 5'-CTCTAGGAGGGCGGAGTCGGA-3', and the mutant probe was 5'-CTCTGAAGAAATAAGACCGGA-3' (only the sense strand is shown). The sequence of the double-stranded probe used for CENPU binding was 5'-AAACCGAAGGTCGGGGACGAC-3', and the mutant probe was 5'-AAACCAGGAACTAAAAACGAC-3' (only the sense strand is shown). During the 20-minute incubation period, the biotin-labelled DNA probes were in contact with the nuclear protein. The protein–DNA complex was electrophoresed on a 6% nondenaturing polyacrylamide gel, transferred to a nylon membrane, and cross-linked for 10–15 minutes with UV light. Finally, biotin-labelled DNA probes were detected using the LightShift Chemiluminescent EMSA Kit (Catalogue Number 20148, Thermo, USA). For the competition experiment, the cold or cold mutated probes were incubated with nuclear protein samples at 30× excess concentrations over the biotin-labelled oligonucleotide concentrations before the addition of labelled probes. For antibody supershift, 4 μl of E2F1 antibody or control IgG was incubated with the reaction mixture at room temperature for 1 h before the addition of the biotin-labelled probe.

Protein stability assay

Cycloheximide (10 μM, Catalogue Number NSC-185, Selleck Chemicals, USA) was added to the cell culture medium to block protein synthesis. Then, protein samples were prepared at the indicated time points and subsequently subjected to western blot analysis.

Ubiquitination assay

Plasmids encoding ubiquitination and E2F6 were cotransfected into HCC cells. Forty-eight hours after transfection, the cells were treated with 10 μM MG132 (Catalogue Number 133407-82-6, MCE, USA) for 4 h. Then, cell lysates were collected and subjected to immunoprecipitation using an anti-His antibody. Finally, the eluted ubiquitinated protein was detected by western blot analysis.

Bioinformatics analysis

The HCC transcriptomic data analysed in the study were downloaded from The Cancer Genome Atlas (TCGA) liver cancer dataset (https://cancergenome.nih.gov/) and Gene Expression Omnibus (GEO) database (accession number GSE40376). Clinical data of HCC patients were downloaded from Kaplan–Meier Plotter (http://kmplot.com/). The differentially expressed genes (DEGs) in the RNA-seq data were screened under the criteria of a FC >2 and a p value <0.05. R software (version 3.6.3) was utilized to process and analyse the DEGs. Single gene set enrichment analysis (GSEA) was performed via GSEA 3.0 (http://www.gsea-msigdb.org/gsea/index.jsp). HitPredict (http://www.hitpredict.org/) was used to predict the interacting protein of CENPU. JASPAR (http://jaspar.genereg.net/) and TRRUST (https://www.grnpedia.org/trrust/) were used to predict the transcription factors of E2F1.

Supplementary Figures

Fig. S1

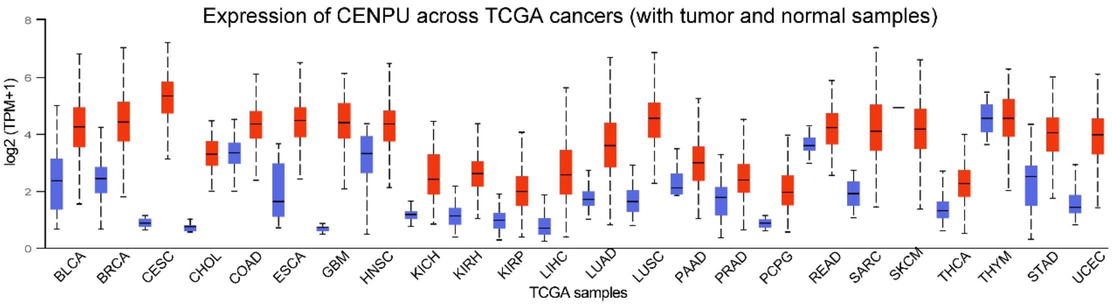

Fig. S1 Transcript levels of CENPU across 24 kinds of cancer types in TCGA.

Fig. S2

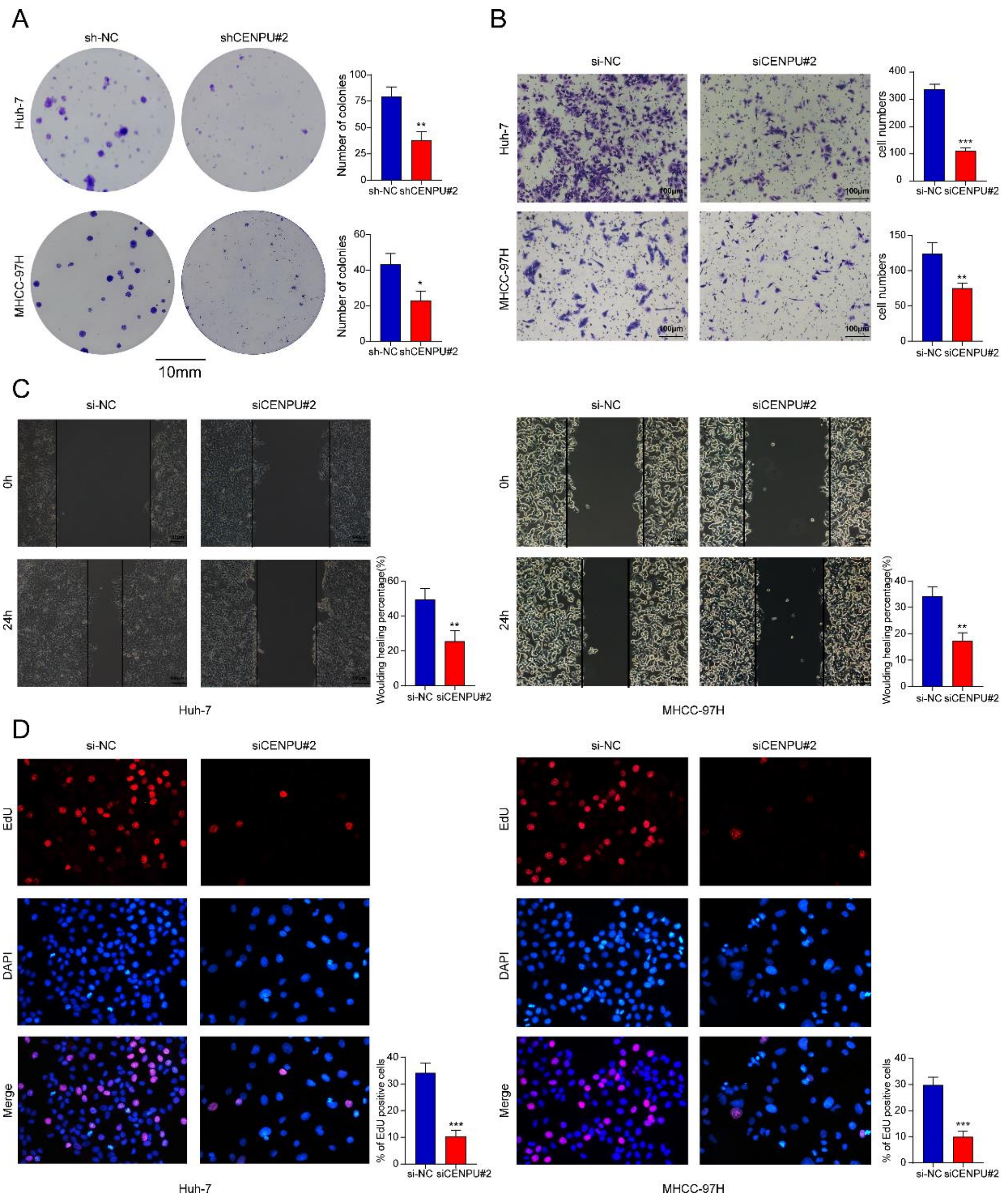

Fig. S2 Depletion of CENPU inhibited the growth, invasion, and migration of HCC cells.

(A) The proliferation ability of hepatoma cells transfected with sh-CENPU or sh-NC was detected by colony formation assays. (B-C) Transwell and wound healing assays of the invasion and migration ability of Huh-7 and MHCC-97H cells following CENPU knockdown. (D) The EdU incorporation assay showed that CENPU downregulation decreased the proportion of cells

entering S phase. $^{**}p < 0.01$; $^{***}p < 0.001$.

Fig. S3

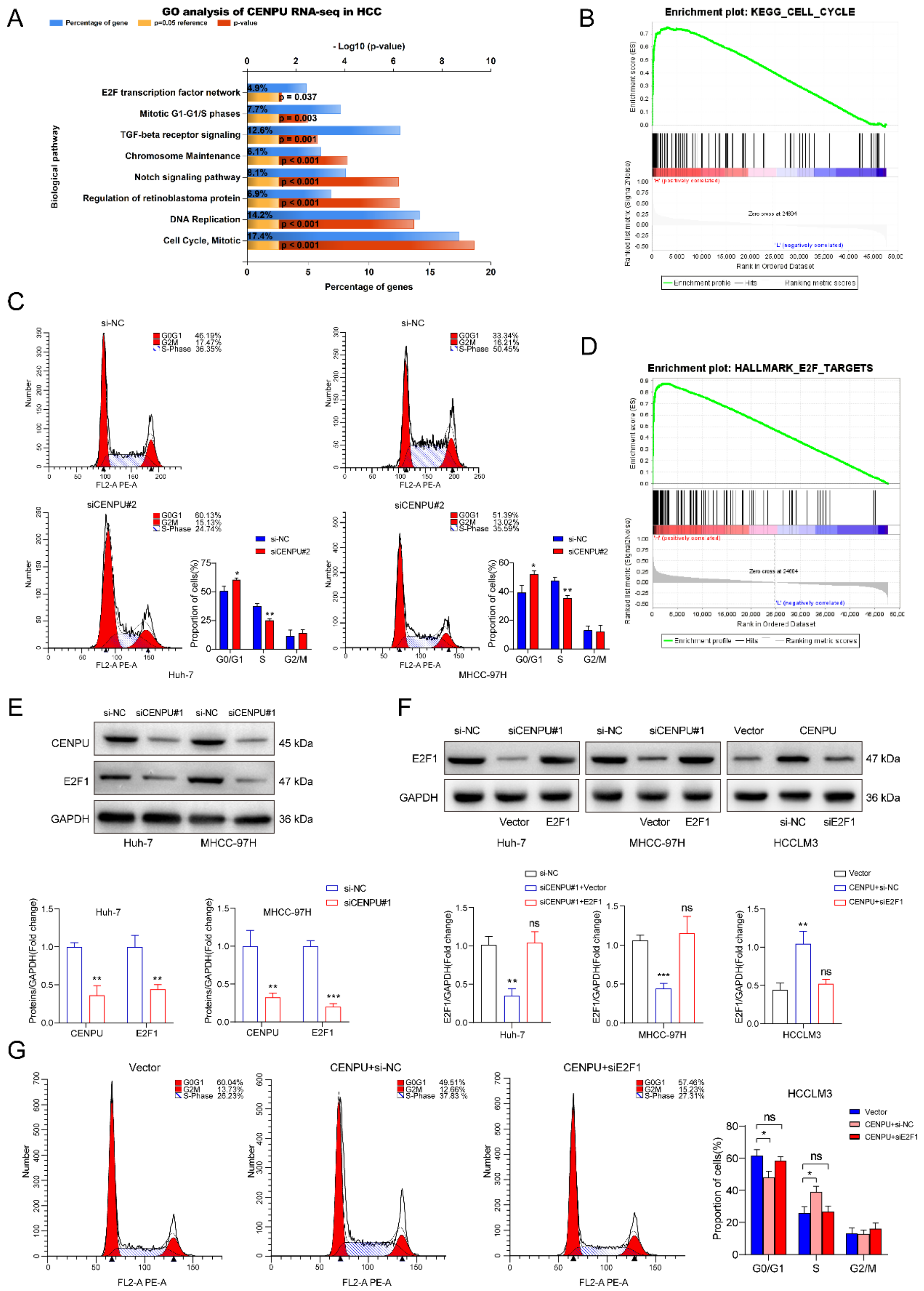

Fig. S3 Upregulation of CENPU accelerated the G1/S transition through E2F1.

(A) GO analysis was performed on the differentially expressed genes from the CENPU RNA-

seq data. (B) GSEA suggested a significant correlation between CENPU and the cell cycle pathway. (C) Flow cytometry analysis showed that CENPU knockdown decreased the proportion of hepatoma cells in S phase. (D) GSEA showed a significant enrichment for the E2F targets pathway based on the high and low expression of CENPU in the TCGA-LIHC database. (E) Immunoblotting analysis of E2F1 expression after CENPU knockdown in Huh-7 and MHCC-97H cells. (F) The efficiency of cotransfection was validated via immunoblotting. (G) Silencing E2F1 counteracted the accelerated G1/S transition induced by CENPU overexpression. ns: no significance; *$p < 0.05$; **$p < 0.01$.

Fig. S4

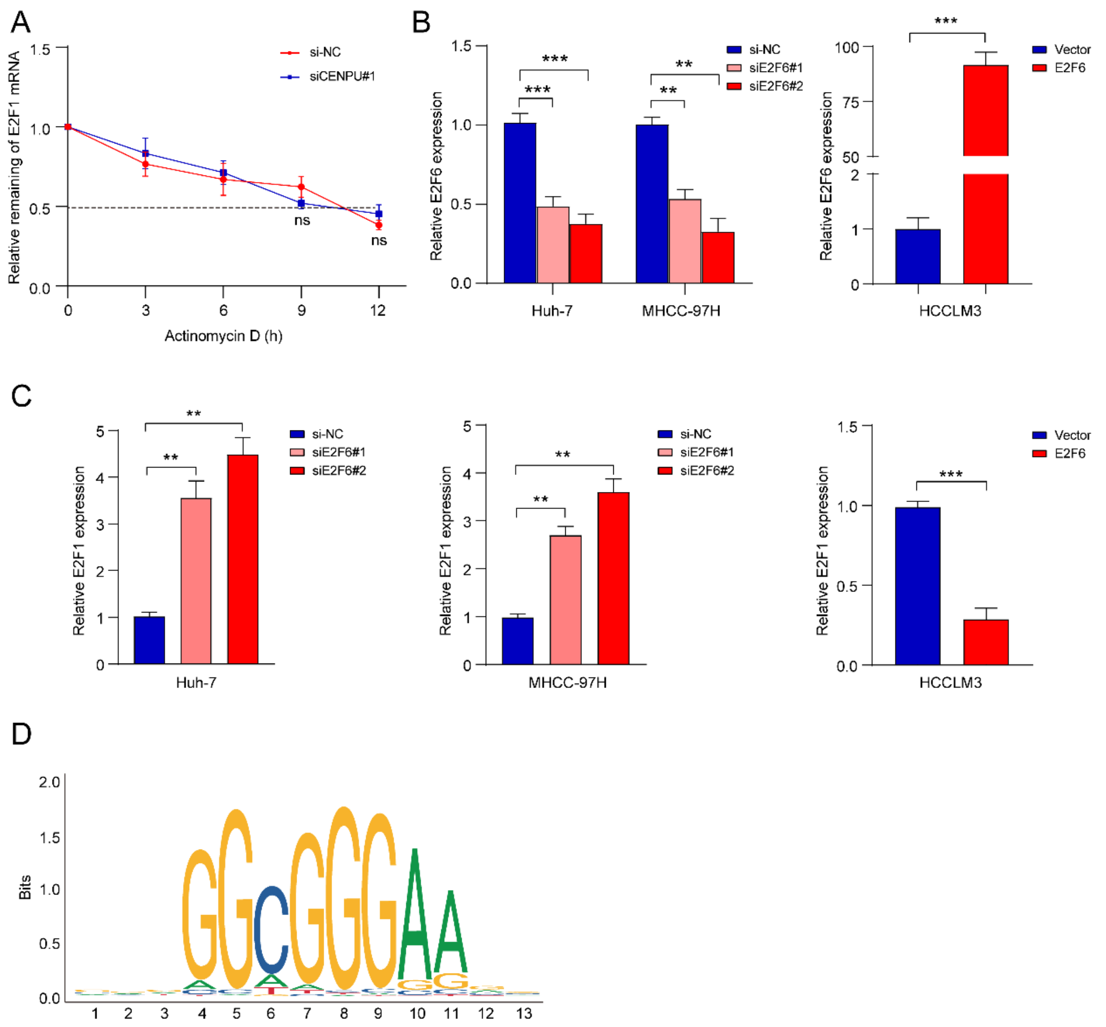

Fig. S4 E2F6 downregulated E2F1 transcriptionally and interacted with CENPU.

(A) RNA stability assays were conducted using actinomycin D to block RNA synthesis in Huh-7 cells, and the degradation rates of E2F1 mRNA were tested by qRT–PCR every 3 h. (B) qRT–PCR analysis of the E2F6 knockdown and overexpression transfection efficiency in HCC cells. (C) The expression of E2F1 was assessed by qRT–PCR analysis in HCC cells transfected with si-E2F6 or E2F6 plasmid. (D) The binding motif of E2F6 predicted by the JASPAR database. **$p < 0.01$; ***$p < 0.001$.

Fig. S5

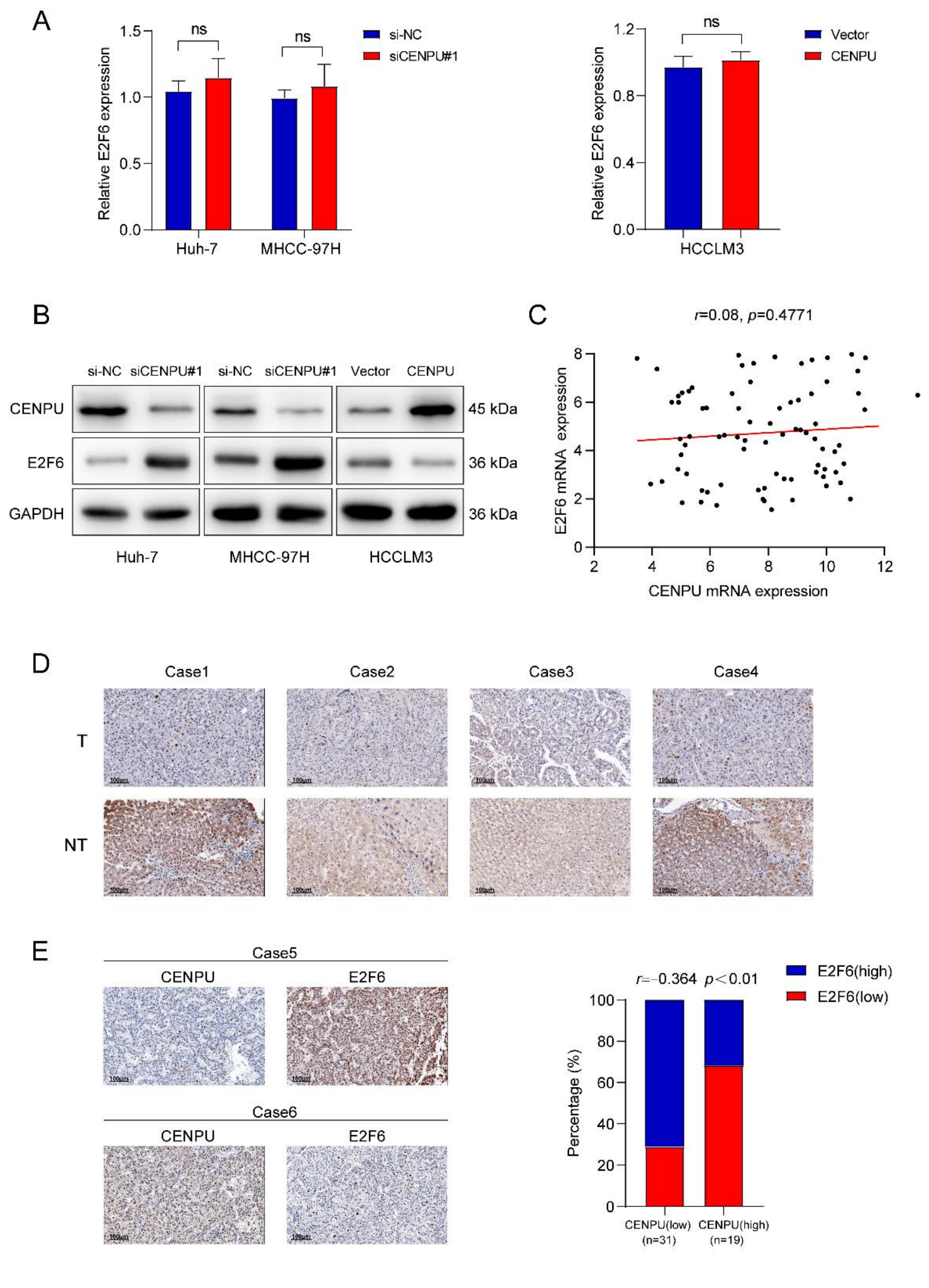

Fig. S5 E2F6 expression was inversely correlated with CENPU expression at the protein level.

(A-B) qRT–PCR and western blot analysis of CENPU and E2F6 expression after CENPU

knockdown or overexpression. (C) Pearson correlation analysis of CENPU and E2F6 mRNA expression in HCC tissues (n=80). (D) E2F6 protein expression in HCC and adjacent nontumor tissues was detected by IHC analysis. (E) Representative results of IHC staining of CENPU and E2F6 in HCC tissues. The association between CENPU and E2F6 expression levels in 50 HCC tissues was investigated. ns: no significance; ** $p < 0.01$; *** $p < 0.001$. T: tumour; NT: nontumor.

Fig. S6

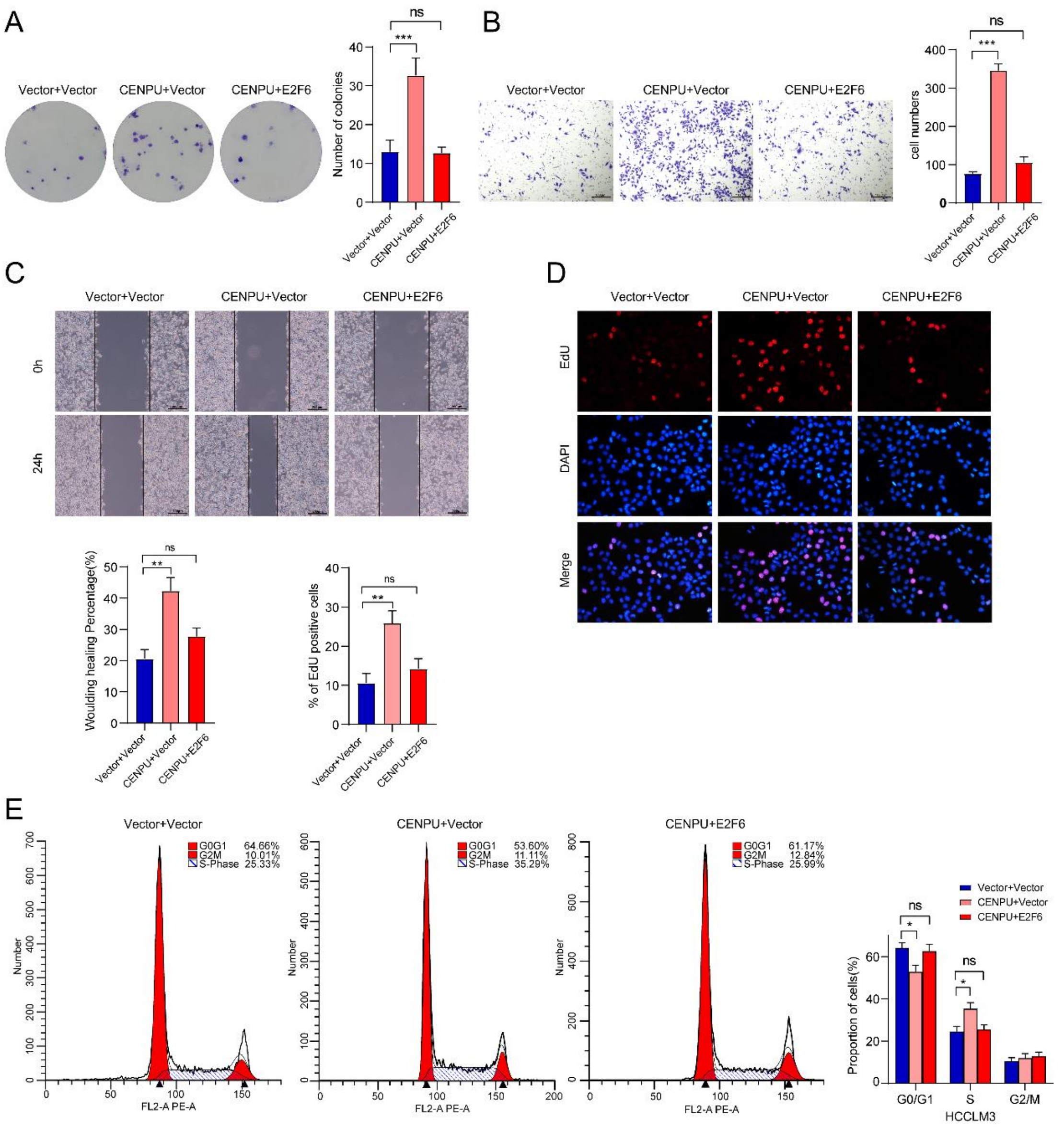

Fig. S6 E2F6 overexpression reversed the impact of CENPU upregulation on the growth, invasion, migration, and cell cycle progression of HCCLM3 cells.

(A) Colony formation assays showing the proliferation ability of HCCLM3 cells cotransfected with CENPU and E2F6 plasmids. (B-C) Transwell and wound healing assays of HCCLM3 cells cotransfected with CENPU and E2F6 plasmids. (D-E) EdU and flow cytometry analysis indicated that ectopic E2F6 could counterbalance the increase in the G1/S transition mediated

by CENPU upregulation. ns: no significance; *$p < 0.05$; **$p < 0.01$; ***$p < 0.001$.

Fig. S7

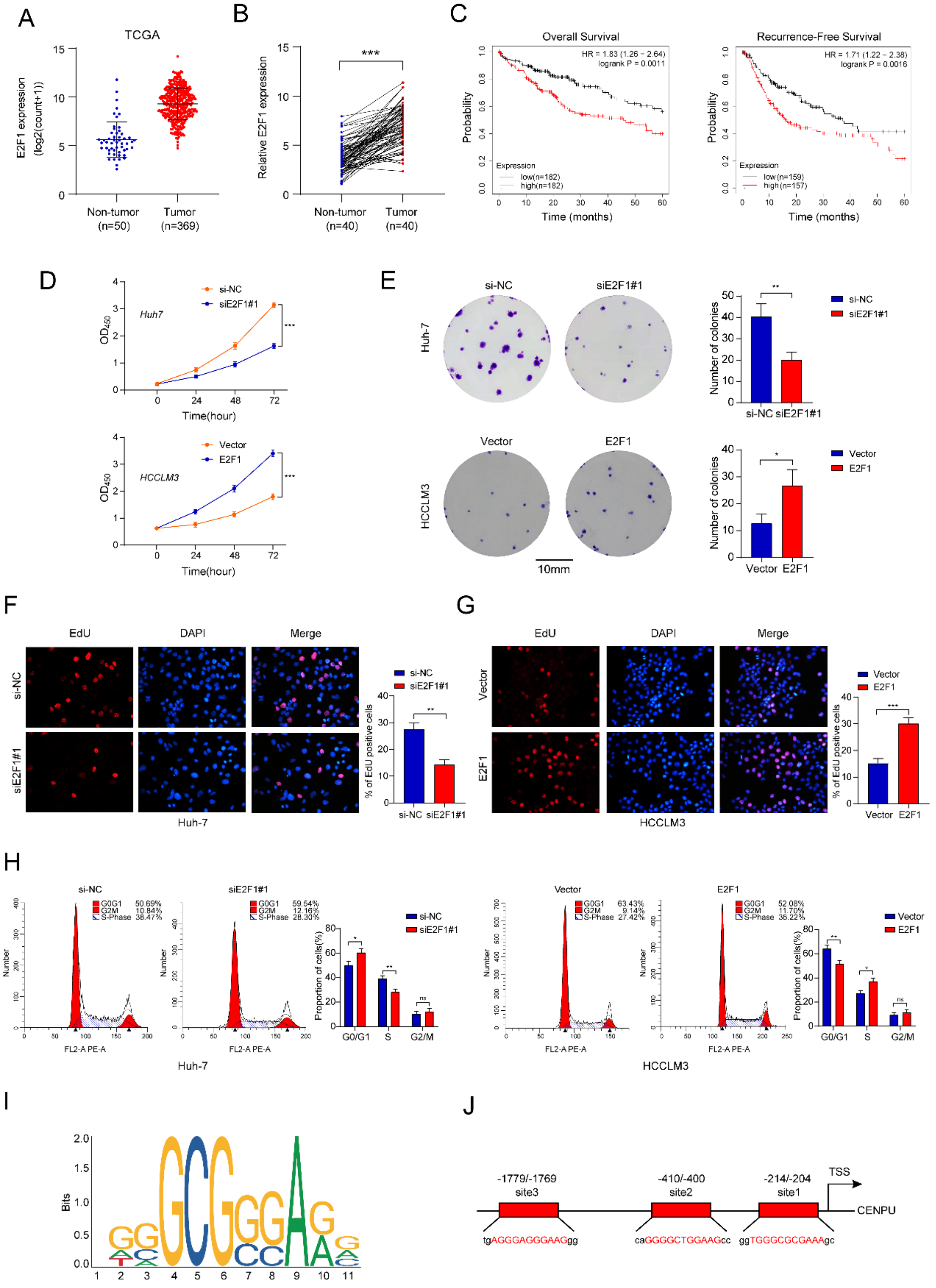

Fig. S7 E2F1 was amplified in HCC and correlated with the malignant phenotype of hepatoma cells.

(A) E2F1 mRNA expression based on the TCGA-LIHC dataset. (B) qRT–PCR analysis of E2F1 expression in 80 paired HCC and nontumor tissues. (C) Kaplan–Meier analysis of the OS and RFS of 364 HCC patients from the TCGA-LIHC database. (D-E) CCK-8 and colony formation assays of Huh-7 and HCCLM3 cells transfected with siE2F1 or E2F1 plasmid. (F-H) EdU and flow cytometry analysis revealed that E2F1 overexpression promoted while E2F1 knockdown blocked the G1/S transition. (I) E2F1-recognizable promoter sequence according to the JASPAR database. (J) Schematic display of the predicted binding sequences of E2F1 on the CENPU promoter region. ns: no significance; *$p < 0.05$; **$p < 0.01$; ***$p < 0.001$.

Table S1. Primers and oligonucleotides sequences

	Interfering oligonucleotides	
PCR primers		Sequence(5'-3')
	CENPU-F	ACCCACCTAGAGCATCAACAA
	CENPU-R	ACTTCAATCATACGCTGCCTTT
	GAPDH-F	AACGCTTCACGAATTTGCGT
	GAPDH-R	GCTGTCACCTTCACCGTTCC
	E2F1-F	ATGTTTTCCTGTGCCCTGAG
	E2F1-R	ATCTGTGGTGAGGGATGAGG
	E2F2-F	GGCCAAGAACAACATCCAGT
	E2F2-R	TGTCCTCAGTCAGGTGCTTG
	E2F3-F	TGAACAAGGCAGCAGAAGTG
	E2F3-R	TTTGACAGGCCTTGACACTG
	E2F4-F	GACCCCACAGGTGTTTTG
	E2F4-R	CCAGGTTGTAGATGTAATCG
	E2F5-F	TCAGGCACCTTCTGGTACAC
	E2F5-R	GGGCTTAGATGAACTCGACTC
	E2F6-F	AGATGCTTTGGATGAGTTA
	E2F6-R	GTTGGTGCTCCTTATGTG
	E2F7-F	CTCCTGTGCCAGAAGTTTC
	E2F7-R	CATAGATGCGTCTCCTTTCC
	E2F8-F	AATATCGTGTTGGCAGAGATCC
	E2F8-R	AGGTTGGCTGTCGGTGTC
siRNAs		
	si-NC	UUCUCCGAACGUGUCACGU
	siCENPU#1	AGCUCAAGAACCAAACGUAAATT
	siCENPU#2	AGACGUUCAAAGAACACUUUATT
	siCENPU#3	ACAUCAAGGAGUUGAAUAUUGTT
	siE2F1#1	GGACCUGGAAACUGACCAUTT
	siE2F1#2	GACCACCUGAUGAAUAUCUTT
	siE2F6#1	GGAAGUUACCCAGUCUCCUTT
	siE2F6#2	AGGAGACUGGGUAACUUCCTT
shRNA		
	sh-NC (CENPU)	TTCTCCGAACGTGTCACGT

sh-CENPU#1	CAGGTATGAGCTATAATAA
sh-CENPU#2	GGAAGATCATTCATAATTT
sh-NC (E2F6)	AGCGCGAGTATACGTGCCTA
sh-E2F6	GCGCTTGGGATCTAGGTTCA

Table S2. Primary antibodies used in this study

Antigens	Manufacturer	Catalog Number	Application
CENPU	Immunoway	YN1585	1:1000 for WB
CENPU	Proteintech	13186-1-AP	1:100 for IHC
			1:50 for IF
GAPDH	Proteintech	60004-1-Ig	1:10000 for WB
E2F1	Abcam	ab94888	1:1000 for WB
			1:100 for IHC
CDK2	Proteintech	10122-1-AP	1:1000 for WB
CDK4	Proteintech	11026-1-AP	1:1000 for WB
			1:500 for IHC
CDK6	Proteintech	14052-1-AP	1:1000 for WB
CCND1	Proteintech	26939-1-AP	1:1000 for WB
CCND1	Abcam	ab40754	1:500 for IHC
CCNE1	Proteintech	11554-1-AP	1:1000 for WB
CCNE1	Abclonal	A14225	1:200 for IHC
CCNB1	Abcam	ab181593	1:2000 for WB
P21	Proteintech	10355-1-AP	1:1000 for WB
MKI67	Proteintech	27309-1-AP	1:5000 for IHC
FLAG-tag	Abcam	ab205606	1:2000 for WB
			1:30 for IP
His-tag	Cell signaling technology	#12698	1:1000 for WB
			1:50 for IP
E2F6	Santa Cruz	sc-390022	1:200 for WB
			1:100 for IF
E2F6	Abclonal	A2718	1:20 for IP
HA-tag	Cell signaling technology	#3724	1:1000 for WB
IgG	Abclonal	AC005	1:200 for IP

Table S3. Kits and reagents used in this study

Kits/Reagents	Manufacturer	Catalog Number	Application
RNAlater	Invitrogen	AM7021	RNA storage
TRIzol	Invitrogen	15596026	RNA extraction
HiScript II Q RT SuperMix	Vazyme	R223-01	Reverse transcription
Taq Pro Universal SYBR qPCR Master Mix	Vazyme	Q712-02	qPCR
Lipofectamine 3000	Invitrogen	L3000015	Transfection
GenMute	SignaGen	SL100568	Transfection
Cell Counting Kit-8	Biosharp	BS350A	CCK8 assay
EdU Staining Proliferation Kit	Abcam	ab222421	EdU assay
Cell cycle staining Kit	MultiSciences	CCS012	Cell cycle analysis
Actinomycin D	Sigma	A4262	RNA stability assay
SimpleChIP Enzymatic Chromatin IP Kit	Cell Signaling Technology	9002S	ChIP
NE-PER Nuclear and Cytoplasmic Extraction Kit	Thermo	78833	Nuclear protein extraction
LightShift Chemiluminescent EMSA Kit	Thermo	20148	EMSA
Cycloheximide	Selleck Chemicals	NSC-185	Protein stability assay
MG132	MCE	133407-82-6	Ubiquitination assay

Table S4. Primers for CHIP analysis

CHIP-PCR primers		Sequence(5'-3')
	E2F1 promoter(-244/-232)	F:AGAAAGGTCAGTGGGATGCG
		R:AGGCTTTGTCCGGATGGTA
	E2F1 promoter(-1632/-1620)	F:TAACCTGGAAGCTTGGTGAGG
		R:TTGCCCAGGGTAGTCTCAAAC
	E2F1 promoter(-1925/-1913)	F:TTATGTGGTCTCTGTGGTCCCTTC
		R:ATGAGGTGGTTTAGGAAATCAGGAG
	CENPU promoter(-410/-400)	F:CAAAAGGAACCCTGTCCATTTAC
		R:CAGGTAAGAGGGTGGGTGGAG

Table S5. Predicted transcription factors of E2F1

HitPredict	JASPAR	TRRUST
CENPP	SP2	ARID3A
CENPO	E2F2	E2F1
PLK1	E2F3	E2F6
CENPQ	E2F4	E2F7
CENPH	E2F7	E2F8
CENPR	E2F6	ELL
CENPI	KLF15	ERCC2
TFP11	KLF5	ESR1
CENPT	ZNF148	HCFC1
CENPN	CTCF	HDAC1
CENPK	SP4	HES1
CENPM	SP1	HIC1
CENPA	ARNT2	IRF1
NUP62	TFEC	NFKB1
CENPC	Arntl	NR0B2
MDC1	ZNF449	NR2F2
ZN512	REL	NR4A1
CENPX	ZNF24	PA2G4
RRP8	ZNF135	PARP1
IMA7	ZBTB6	PAX8
RL10	ZNF460	PGR
RBM34	IRF1	POLR1A
RL28	ZKSCAN5	RB1
CENPF	SPIB	RELA
CNBP	ELF1	RORA
RL10A	GABPA	SIN3A
RL18A	EHF	SP1
GOGA3	KLF9	TBP
ZMYM1	MYCN	TFDP3
SPT2	Stat2	TP53
MIER1	ZNF384	TP73
DGCR8	ATF6	TRIM16
ZNF2	CREB3L1	YBX1
RBM4	GFL1	
ZNF71	GCM1	
FND3A	DMRT3	
NS1BP		
MLF1		
SART3		
PLXB1		
NHS		

NDUS2
XRCC6
IF4G1
TBB2B
SPTN4
GIT1
PAXI1
PLXB2
E2F6
RENT2
VIME
CENPL
CENPS
TAU
PLK3
PLK2
SI1L1
FGF3
KNOP1
MET2B
MITD1
PGRC1
LAMC1
ROBO2

Table S6. Predicted transcription factors of CENPU

**UCSC**
ZNF93
EGR1
ZNF682
Nrf1
GCM1
GCM2
ZNF454
MYCN
KLF17
ZNF263
HSF1
HSF2
CUX1
Nfat5
FOXD3
Runx1
ONECUT1
PBX2
TCF4
NFATC3
ASCL1
ZNF384
MEF2A
MEF2D
Nr1H4
Nr2F6
TBP
E2F1
ZBTB26
TFAP2A
GATA2
LEF1
SOX4
SP4
KLF5
STAT3
ZEB1
TEAD1
IRF1
MAFF
Zfx

ETV5
NHLH2
MZF1
Rhox11
GRHL2

Table S7. E2F6 potential E3 ligase from Ubibrowse

E3	E3GENE	SUBGENE	HOMO	PFAM	GO	NET	MOTIF	SCORE
Q9HCE7	SMURF1	E2F6	1	1	2.88	1.29	2.12	0.71
Q9HAU4	SMURF2	E2F6	1	1	3.98	1.77	1.06	0.705
Q00987	MDM2	E2F6	1	1	4.05	1.69	1.06	0.703
Q9UM11	FZR1	E2F6	1	1	2.33	1.29	2.12	0.691
Q9UNE7	STUB1	E2F6	1	1	3.98	1.29	1	0.671
Q86TM6	SYVN1	E2F6	1	1	1.25	1.44	2.8	0.669
Q13309	SKP2	E2F6	1	1	2.33	2.3	1.06	0.655
Q8WY64	MYLIP	E2F6	1	1	1.25	1	2.8	0.633
Q5T0T0	8-Mar	E2F6	1	1	1.25	1	2.8	0.633
O43684	BUB3	E2F6	1	1	2.33	1.44	1	0.628
Q12834	CDC20	E2F6	1	1	2.33	1.44	1	0.628
Q86YT6	MIB1	E2F6	1	1	1.51	1	2.12	0.624
P51668	UBE2D1	E2F6	1	1	2.33	1.29	1	0.617
Q96Q27	ASB2	E2F6	1	1	1	1	2.8	0.61
Q8N3Y1	FBXW8	E2F6	1	1	1	1	2.8	0.61
Q9HC52	CBX8	E2F6	1	1	1.51	2.17	1	0.609
P14373	TRIM27	E2F6	1	1	1.51	2.39	1	0.609
O00257	CBX4	E2F6	1	1	1.51	2.39	1	0.609
P51948	MNAT1	E2F6	1	1	1.51	2.39	1	0.609
Q99728	BARD1	E2F6	1	1	1.51	1.77	1	0.605
P63244	GNB2L1	E2F6	1	1	1.51	1.77	1	0.605
Q66K89	E4F1	E2F6	1	1	1.51	1.77	1	0.605
Q92466	DDB2	E2F6	1	1	1.51	2.3	1	0.605
Q92831	KAT2B	E2F6	1	1	1.51	1.77	1	0.605
P35227	PCGF2	E2F6	1	1	1.51	1.77	1	0.605
Q9NWF9	RNF216	E2F6	1	1	1.25	1	2.12	0.604
Q14258	TRIM25	E2F6	1	1	1.25	1	2.12	0.604
Q86YJ5	9-Mar	E2F6	1	1	1.25	1	2.12	0.604
Q15386	UBE3C	E2F6	1	1	1.25	1	2.12	0.604
Q8TCQ1	1-Mar	E2F6	1	1	1.25	1	2.12	0.604
P53804	TTC3	E2F6	1	1	1.25	1	2.12	0.604
